# Supplementary material for: Health-related quality of life and prevalence of six chronic diseases in homeless and housed people: a cross-sectional study in London and Birmingham, England
Source: BMJ Open. 2019 Apr 24;9(4):e025192. doi: 10.1136/bmjopen-2018-025192 (PMC6501971; doi:10.1136/bmjopen-2018-025192)
Supplement: Supplementary file 1 [file bmjopen-2018-025192supp001.pdf]

Supplementary information

1. Table S1: prevalence of long term conditions by age group
2. Figure. Health-related quality of life in participants with one or more long-term condition
3. Table S2: Sensitivity analysis excluding Health Survey for England data 2006 and 2008
4. Table S3: QALY scores, visual-analogue method
5. Table S4: QALY scores, time trade-off method

1. Table S1. prevalence of long-term condition by age group: cases/sample size (prevalence; 95% confidence intervals). Stroke is excluded to avoid publication of small numbers

Condition	Age group	Find & Treat (homeless)	Health Survey for England (housed)
Asthma	16-34	88/504 (17.5; 14.3-21.1)	300/5040 (6.0; 5.3-6.7)
	35-44	76/373 (20.4; 16.5-24.9)	232/3740 (6.2; 5.5-7.0)
	45-54	52/315 (16.5; 12.7-21.2)	164/3150 (5.2; 4.5-6.1)
	55-64	28/143 (19.6; 13.6-27.2)	60/1430 (4.2; 3.2-5.4)
		p=0.542	p=0.020
COPD	16-34	48/504 (9.5; 7.2-12.5)	23/5040 (0.5; 0.3-0.7)
	35-44	50/374 (13.4; 10.2-17.3)	36/3740 (1.0; 0.7-1.3)
	45-54	50/315 (15.9; 12.1-20.5)	60/3150 (1.9; 1.5-2.5)
	55-64	39/143 (27.3; 20.3-35.5)	61/1430 (4.3; 3.3-5.5)
		p<0.001	p<0.001
Epilepsy	16-34	23/504 (4.6; 3.0-6.9)	38/5040 (0.8; 0.5-1.0)
	35-44	26/374 (7.0; 4.7-10.1)	30/3740 (0.8; 0.6-1.2)
	45-54	23/315 (7.3; 4.8-10.9)	30/3150 (1.0; 0.7-1.4)
	55-64	8/143 (5.6; 2.6-11.1)	10/1430 (0.7; 0.4-1.3)
		p=0.329	p=0.749
Heart problems	16-34	16/504 (3.2; 1.9-5.2)	24/5040 (0.5; 0.3-0.7)
	35-44	25/374 (6.7; 4.5-9.8)	42/3740 (1.1; 0.8-1.5)
	45-54	36/315 (11.4; 8.2-15.6)	107/3150 (3.4; 2.8-4.1)
	55-64	26/143 (18.2; 12.4-25.7)	93/1430 (6.5; 5.3-7.9)
		p<0.001	p<0.001
Diabetes	16-34	8/504 (1.6; 0.7-3.2)	42/5040 (0.8; 0.6-1.1)
	35-44	14/374 (3.7; 2.1-6.3)	88/3740 (2.4; 1.9-2.9)
	45-54	22/315 (7.0; 4.5-10.5)	172/3150 (5.5; 4.7-6.3)
	55-64	11/143 (7.7; 4.1-13.7)	129/1430 (9.0; 7.6-10.7)
		p<0.001	p<0.001

Age groups 16-24 and 25-34 have been merged to avoid publication of small numbers. p-values are based on chi-square tests.

2. Figure S1. Odds ratios of reported problems in EQ-5D-3L domains (some and severe problems grouped), by deprivation and presence of any long-term conditions, with 95% confidence intervals

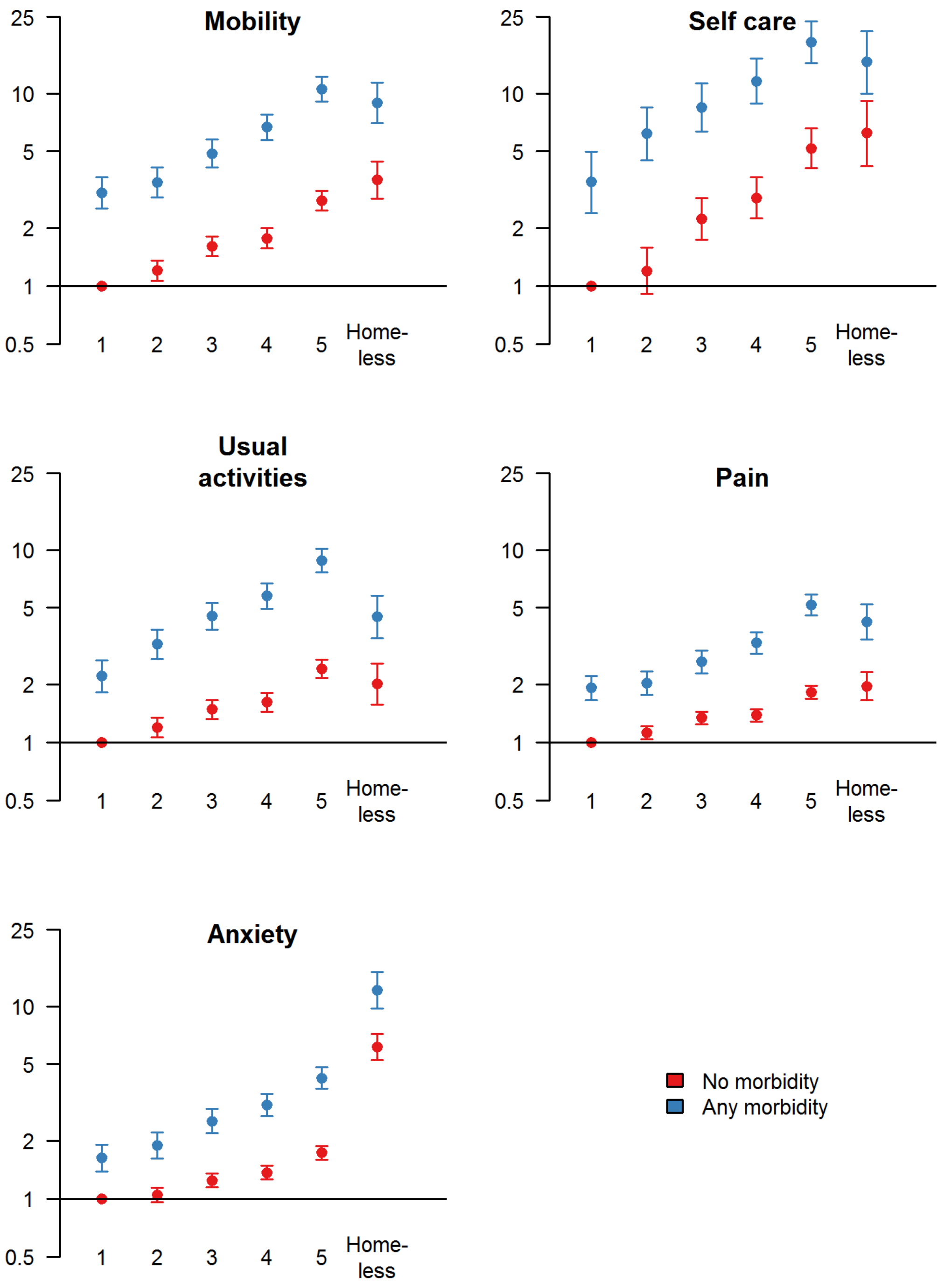

x-axis show deprivation groups (1 = least deprived quintile of Index of Multiple Deprivation; 5 = most deprived quintile of Index of Multiple deprivation). Y-axis shows odds ratios, with least deprived group with no morbidity as the reference group.

3. Table S2: Sensitivity analysis excluding Health Survey for England data 2006 and 2008: prevalence ratio comparing homeless participants to the least deprived housed quintile

		Original analysis	Excluding HSE data from 2006 and 2008
**Long-term conditions**	Asthma	3.3 (2.8-4.1)	3.7 (2.8-4.7)
	Breathing	12.9 (8.8-19.0)	16.6 (9.3-29.5)
	Epilepsy	10.7 (6.2-18.4)	12.1 (5.6-26.2)
	Heart problems	5.1 (3.6-7.4)	6.8 (4.1-11.5)
	Stroke	6.9 (3.0-15.9)	8.5 (2.6-28.2)
	Diabetes	1.4 (1.0-2.0)	1.6 (1.1-2.5)
**EQ-5D problems**	Mobility	3.5 (2.9-4.3)	3.3 (2.6-4.2)
	Self-care	6.1 (4.1-9.1)	7.6 (4.3-13.5)
	Usual activities	2.2 (1.8-2.7)	2.0 (1.6-2.5)
	Pain	1.7 (1.5-1.9)	1.6 (1.4-1.8)
	Anxiety	3.8 (3.4-4.2)	3.2 (2.8-3.7)

4. Table S3: QALY scores, visual analogue method

					Mean				Quantiles				
Group	Sex	Age	N	Missing	Mean	sd	0.025 CI	0.975 CI	0%	25%	50%	75%	100%
Homeless	Female	16-24	46	6	0.736	0.207	0.670	0.803	0.351	0.506	0.761	1.000	1.000
		25-34	69	5	0.687	0.240	0.627	0.747	0.233	0.505	0.725	1.000	1.000
		35-44	69	7	0.634	0.260	0.568	0.700	0.196	0.435	0.694	0.782	1.000
		45-54	52	8	0.614	0.274	0.531	0.698	-0.073	0.432	0.675	0.782	1.000
		55-64	13	3	0.593	0.259	0.407	0.779	0.227	0.350	0.669	0.769	1.000
	Male	16-24	94	10	0.816	0.209	0.771	0.862	0.270	0.698	0.798	1.000	1.000
		25-34	295	33	0.778	0.227	0.750	0.806	0.140	0.673	0.782	1.000	1.000
		35-44	305	32	0.720	0.240	0.691	0.748	0.038	0.506	0.761	1.000	1.000
		45-54	263	26	0.701	0.264	0.667	0.734	0.138	0.503	0.761	1.000	1.000
		55-64	130	12	0.704	0.270	0.655	0.753	-0.023	0.506	0.761	1.000	1.000
Housed	Female	16-24	460	45	0.914	0.147	0.900	0.928	0.151	0.782	1.000	1.000	1.000
		25-34	690	63	0.908	0.155	0.896	0.921	0.140	0.782	1.000	1.000	1.000
		35-44	690	50	0.877	0.180	0.863	0.891	0.090	0.761	1.000	1.000	1.000
		45-54	520	56	0.819	0.217	0.800	0.839	-0.073	0.698	0.782	1.000	1.000
		55-64	130	4	0.811	0.222	0.772	0.851	0.029	0.692	0.782	1.000	1.000
	Male	16-24	940	112	0.937	0.132	0.928	0.946	0.151	1.000	1.000	1.000	1.000
		25-34	2950	321	0.921	0.146	0.915	0.926	-0.021	0.782	1.000	1.000	1.000
		35-44	3050	317	0.889	0.175	0.882	0.895	0.038	0.761	1.000	1.000	1.000
		45-54	2630	256	0.862	0.197	0.854	0.870	-0.073	0.761	1.000	1.000	1.000
		55-64	1300	129	0.815	0.226	0.802	0.828	0.075	0.698	0.782	1.000	1.000
Homeless	Female	All	249	29	0.662	0.250	0.629	0.696	-0.073	0.503	0.698	0.782	1.000
	Male	All	1087	113	0.737	0.247	0.722	0.753	-0.023	0.506	0.778	1.000	1.000
Housed	Female	All	2490	218	0.877	0.183	0.870	0.885	-0.073	0.761	1.000	1.000	1.000
	Male	All	10870	1135	0.886	0.181	0.882	0.890	-0.073	0.761	1.000	1.000	1.000
Homeless	Both	16-24	140	16	0.791	0.211	0.753	0.828	0.270	0.677	0.782	1.000	1.000
		25-34	364	38	0.760	0.232	0.735	0.785	0.140	0.523	0.782	1.000	1.000
		35-44	374	39	0.704	0.246	0.677	0.730	0.038	0.506	0.761	1.000	1.000
		45-54	315	34	0.687	0.267	0.656	0.718	-0.073	0.475	0.761	1.000	1.000
		55-64	143	15	0.695	0.270	0.648	0.742	-0.023	0.506	0.746	1.000	1.000
Housed	Both	16-24	1400	157	0.929	0.137	0.922	0.937	0.151	1.000	1.000	1.000	1.000
		25-34	3640	384	0.918	0.148	0.913	0.924	-0.021	0.782	1.000	1.000	1.000
		35-44	3740	367	0.887	0.176	0.881	0.893	0.038	0.761	1.000	1.000	1.000
		45-54	3150	312	0.855	0.201	0.848	0.862	-0.073	0.761	1.000	1.000	1.000
		55-64	1430	133	0.814	0.226	0.802	0.827	0.029	0.698	0.782	1.000	1.000
Homeless	Both	All	1336	142	0.723	0.249	0.709	0.737	-0.073	0.506	0.761	1.000	1.000
Housed	Both	All	13360	1353	0.884	0.181	0.881	0.888	-0.073	0.761	1.000	1.000	1.000

5. Table S3: QALY scores, time trade-off method

					Mean				Quantiles				
**Group**	**Sex**	**Age**	**N**	**Missing**	**Mean**	**sd**	**0.025 CI**	**0.975 CI**	**0%**	**25%**	**50%**	**75%**	**100%**
Homeless	Female	16-24	46	6	0.732	0.250	0.652	0.811	0.222	0.491	0.796	1.000	1.000
		25-34	69	5	0.656	0.311	0.579	0.734	-0.077	0.414	0.770	1.000	1.000
		35-44	69	7	0.583	0.358	0.492	0.674	-0.077	0.305	0.726	0.848	1.000
		45-54	52	8	0.561	0.399	0.439	0.682	-0.594	0.291	0.708	0.848	1.000
		55-64	13	3	0.571	0.337	0.330	0.812	0.082	0.233	0.708	0.831	1.000
	Male	16-24	94	10	0.813	0.244	0.760	0.866	0.128	0.725	0.866	1.000	1.000
		25-34	295	33	0.769	0.282	0.734	0.803	-0.181	0.700	0.848	1.000	1.000
		35-44	305	32	0.702	0.308	0.665	0.739	-0.349	0.414	0.796	1.000	1.000
		45-54	263	26	0.668	0.343	0.624	0.712	-0.181	0.414	0.796	1.000	1.000
		55-64	130	12	0.668	0.361	0.602	0.734	-0.536	0.414	0.796	1.000	1.000
Housed	Female	16-24	460	45	0.924	0.150	0.909	0.938	-0.074	0.848	1.000	1.000	1.000
		25-34	690	63	0.916	0.166	0.903	0.929	-0.181	0.848	1.000	1.000	1.000
		35-44	690	50	0.883	0.203	0.867	0.898	-0.239	0.796	1.000	1.000	1.000
		45-54	520	56	0.822	0.260	0.798	0.845	-0.594	0.727	0.848	1.000	1.000
		55-64	130	4	0.810	0.272	0.762	0.858	-0.426	0.727	0.848	1.000	1.000
	Male	16-24	940	112	0.944	0.134	0.935	0.953	-0.074	1.000	1.000	1.000	1.000
		25-34	2950	321	0.928	0.153	0.922	0.934	-0.484	0.848	1.000	1.000	1.000
		35-44	3050	317	0.895	0.193	0.888	0.902	-0.358	0.796	1.000	1.000	1.000
		45-54	2630	256	0.865	0.226	0.856	0.874	-0.594	0.796	1.000	1.000	1.000
		55-64	1300	129	0.814	0.268	0.798	0.829	-0.239	0.727	0.850	1.000	1.000
Homeless	Female	All	249	29	0.626	0.338	0.581	0.671	-0.594	0.414	0.727	0.848	1.000
	Male	All	1087	113	0.717	0.316	0.697	0.737	-0.536	0.414	0.848	1.000	1.000
Housed	Female	All	2490	218	0.883	0.207	0.874	0.891	-0.594	0.796	1.000	1.000	1.000
	Male	All	10870	1135	0.891	0.202	0.887	0.895	-0.594	0.796	1.000	1.000	1.000
Homeless	Both	16-24	140	16	0.786	0.248	0.742	0.831	0.128	0.721	0.848	1.000	1.000
		25-34	364	38	0.747	0.291	0.715	0.778	-0.181	0.533	0.848	1.000	1.000
		35-44	374	39	0.680	0.321	0.645	0.714	-0.349	0.414	0.796	1.000	1.000
		45-54	315	34	0.651	0.354	0.610	0.693	-0.594	0.378	0.796	1.000	1.000
		55-64	143	15	0.660	0.359	0.597	0.723	-0.536	0.414	0.788	1.000	1.000
Housed	Both	16-24	1400	157	0.937	0.140	0.930	0.945	-0.074	1.000	1.000	1.000	1.000
		25-34	3640	384	0.926	0.155	0.920	0.931	-0.484	0.848	1.000	1.000	1.000
		35-44	3740	367	0.893	0.195	0.886	0.899	-0.358	0.796	1.000	1.000	1.000
		45-54	3150	312	0.858	0.232	0.849	0.867	-0.594	0.796	1.000	1.000	1.000
		55-64	1430	133	0.813	0.269	0.799	0.828	-0.426	0.727	0.850	1.000	1.000
Homeless	Both	All	1336	142	0.700	0.322	0.682	0.719	-0.594	0.414	0.812	1.000	1.000
Housed	Both	All	13360	1353	0.890	0.203	0.886	0.893	-0.594	0.796	1.000	1.000	1.000
