# Supplementary material for: Health-related quality of life and prevalence of six chronic diseases in homeless and housed people: a cross-sectional study in London and Birmingham, England
Source: BMJ Open. 2019 Apr 24;9(4):e025192. doi: 10.1136/bmjopen-2018-025192 (PMC6501971; doi:10.1136/bmjopen-2018-025192)
Supplement: Author's manuscript [file bmjopen-2018-025192.draft_revisions.pdf]

BMJ Open

BMJ Open is committed to open peer review. As part of this commitment we make the peer review history of every article we publish publicly available.

When an article is published we post the peer reviewers' comments and the authors' responses online. We also post the versions of the paper that were used during peer review. These are the versions that the peer review comments apply to.

The versions of the paper that follow are the versions that were submitted during the peer review process. They are not the versions of record or the final published versions. They should not be cited or distributed as the published version of this manuscript.

BMJ Open is an open access journal and the full, final, typeset and author-corrected version of record of the manuscript is available on our site with no access controls, subscription charges or pay-per-view fees (http://bmjopen.bmj.com).

If you have any questions on BMJ Open's open peer review process please email info.bmjopen@bmj.com

BMJ Open

Slopes and cliffs in health inequalities: a cross-sectional study comparing the health of homeless and housed populations of England

Journal:	*BMJ Open*
Manuscript ID	bmjopen-2018-025192
Article Type:	Research
Date Submitted by the Author:	04-Jul-2018

Complete List of Authors:	Lewer, Dan; University College London, Institute of Epidemiology and Healthcare<br>Aldridge, Robert; University College London, Farr Institute of Health Informatics Research<br>Menezes, Dee; University College London, UCL Farr Institute of Health Informatics Research<br>Sawyer, Clare; University College London Hospitals NHS Foundation Trust, Find & Treat<br>Dedicoat, Martin; Heart of England NHS Foundation Trust<br>Ahmed, Imtiaz; Sandwell and West Birmingham Hospitals NHS Trust<br>Luchenski, Serena; University College London, UCL Farr Institute of Health Informatics Research<br>Hayward, Andrew; University College London, Infection and Population Health<br>Story, Alistair; University College London Hospitals NHS Foundation Trust, Find & Treat

Keywords:	PUBLIC HEALTH, EPIDEMIOLOGY, Homelessness, Inequality

SCHOLARONE™
Manuscripts

Slopes and cliffs in health inequalities: a cross-sectional study comparing the health of homeless and housed populations of England

Authors

Dan Lewer (MSc)[1,2], Robert W Aldridge (PhD)[1], Dee Menezes (MPH) [1], Clare Sawyer (MSc)[3], Martin J Dedicoat (PhD)[4], Imtiaz Ahmed (FRCP)[5], Serena A Luckenski (FFPH)[1], Andrew Hayward (MD)[2]*, Alistair Story (PhD)[2,3]*

*Joint senior authors

Affiliations

1. Institute of Health Informatics, University College London, 222 Euston Road, London NW1 2DA, United Kingdom
2. Institute of Epidemiology and Healthcare, University College London, 1-19 Torrington Place, London WC1E 6BT, United Kingdom
3. Find and Treat, University College London Hospitals NHS Foundation Trust, 250 Euston Road, London NW1 2PG, United Kingdom
4. Heart of England NHS Foundation Trust, Bordesley Green East, Birmingham B9 5SS, United Kingdom
5. Sandwell and West Birmingham Hospitals NHS Trust, Sandwell General Hospital, West Bromwich B71 4HJ, United Kingdom

Corresponding author

Dan Lewer, d.lewer@ucl.ac.uk, +44 (0) 203 549 5142

Word count: 2,925

For peer review only

Abstract

Objectives: To compare the prevalence of chronic diseases and health-related quality of life in housed and homeless populations.

Design: Cross-sectional survey of homeless people, with a housed comparison group drawn from the Health Survey for England. Housed participants were grouped according to their neighbourhood deprivation quintile. To account for differences in age and sex, we calculated directly adjusted prevalences and standardised prevalence ratios.

Setting: Hostels, day centres and soup runs in London and Birmingham, England.

Participants: All individuals attending the services were eligible to participate. The study included 1336 homeless and 36673 housed participants.

Outcome measures: Chronic diseases were self-reported asthma, chronic obstructive pulmonary disease, epilepsy, heart problems, stroke, and diabetes. Health-related quality of life was measured using EQ-5D-3L.

Results: Social gradients were observed in the housed population for all diseases, in which participants in more deprived neighbourhoods were more likely to report disease. Homeless participants were substantially more likely than housed participants in any deprivation quintile to report all diseases except diabetes (which had similar prevalence in homeless participants and the most deprived housed group). For example, the adjusted prevalence of chronic obstructive pulmonary disease was 0.8% (95% CI 0.6%, 1.1%) in the least deprived housed quintile; 2.3% (95% CI 1.8%, 2.7%) in the most deprived housed quintile; and 14.0% (95% CI 12.2%, 15.8%) in the homeless group. Social gradients were also seen for problems in each EQ-5D-3L domain in the housed population, but homeless participants had similar likelihood of reporting problems as the most deprived housed group. The exception was problems related to anxiety, which were substantially more common in homeless people than any of the housed groups.

Conclusions: While differences in health between housed socioeconomic groups can be described as a 'slope', differences in health between housed and homeless people are better understood as a 'cliff'.

For peer review only - http://bmjopen.bmj.com/site/about/guidelines.xhtml

STRENGTHS AND LIMITATIONS OF THIS STUDY

- This study provides one of the first attempts to investigate common long-term conditions in homeless people.
- Most existing studies of morbidity in homeless people focus on mental health and infectious disease, while this study includes a range of common long-term conditions such as respiratory and circulatory diseases.
- The study uses a housed comparison group and shows how differences between housed and homeless people compare to inequalities within the housed population.
- Causes of differences in health (such as lifestyle factors) are not investigated.
- All data are collected through self-report.

INTRODUCTION

Inequalities in disease prevalence in the general population are widely observed[1,2] but few studies have looked at morbidity in highly marginalised groups and there are few comparisons with the general population.[3] Existing studies show that homeless people have worse health than those in stable housing,[4–8] focusing on high relative rates of mental health problems and infectious diseases.[9]

Studies of health inequalities often exclude these experiences because common measures of socioeconomic class, such as occupation and neighbourhood deprivation, do not capture extreme social exclusion. In addition, large population health surveys typically sample people living in private households, excluding groups such as prisoners and homeless people. 'Slopes' of inequality have been observed for almost all health outcomes and risk factors, in which those with lower socioeconomic status have worse health and more risk factors. However, highly marginalised groups may have substantially worse health and, in a sense, fall off the slope of inequality.

Our aim was to describe inequality in the prevalence of chronic disease in the housed population of England (across quintiles of neighbourhood deprivation) and compare this to homeless groups. We expected chronic diseases to be more common in the homeless group than in any of the housed groups.

METHODS

Data sources

We undertook a cross-sectional health survey of homeless people alongside a mobile tuberculosis screening unit at hostels, day centres and soup runs. Questions were tested with homeless people before the fieldwork. Interviewer-led questionnaires were conducted in London from 1 July 2012 to 31 August 2012 and in Birmingham from 13 July 2014 to 18 July 2014 and from 16 March 2015 to 24 July 2015. All clients at

For peer review only

For peer review only - http://bmjopen.bmj.com/site/about/guidelines.xhtml

the location were eligible to participate (i.e. the survey was not limited to people attending screening) and verbal consent was obtained. Interviewers were trained. Participants aged under 16 or over 64 were excluded from this analysis due to small numbers. Research ethics was not required because the survey was conducted as part of a service evaluation and anonymised data was used in this analysis.

We selected a housed comparison group from the Health Survey for England; an annual series of cross-sectional surveys designed to be representative of adults living in private households.[10] We used data from the 2008, 2010, 2011, 2012 and 2014 surveys, aged 16-64 and from urban areas, as the surveys of homeless people were conducted in urban areas.

Variables

The main outcomes are self-reported long-term conditions and health-related quality of life.

To establish prevalence of long-term conditions, homeless participants were asked whether a doctor or nurse had ever given a diagnosis of (i) asthma, (ii) bronchitis, emphysema or obstructive airways disease (abbreviated to COPD, chronic obstructive pulmonary disease), (iii) epilepsy, (iv) heart problems, including heart attack, angina, murmur or abnormal heart rhythms, (v) stroke, and (vi) diabetes. Housed participants were asked whether they had a health condition lasting or expected to last 12 months or more, with corresponding options of (i) asthma, (ii) bronchitis, emphysema or other respiratory complaints, (iii) epilepsy, fits or convulsions, (iv) heart attack, angina or other heart problems, (v) stroke, cerebral haemorrhage or cerebral thrombosis and (vi) diabetes including hyperglycaemia.

EQ-5D-3L[11] was used to measure health-related quality of life. All participants considered whether they had no problems, some problems or extreme problems with mobility, pain, self-care, usual activities and anxiety. ‘Some problems’ and ‘extreme problems’ were grouped. Participants also completed a visual-analogue scale of overall health state on the day of interview, where 0 is the worst imaginable state and 100 the best.

The main exposure was deprivation. The homeless group were classified as the most deprived, while the housed comparison group were classified using quintiles of the Index of Multiple Deprivation 2007.[12] This is an area-based index derived from levels of income, employment, health, disability, education, skills and training in small local areas of roughly 1500 residents.

Age was grouped into 16-24, 25-34, 35-44, 45-54 and 55-64, which was consistently available across datasets.

Country of origin was collected for the homeless group, but was not available for the housed comparison group. We therefore used data from the UK’s Census 2011 (limiting to those living in urban areas aged 16-64) as an estimate of the proportion of the housed group from each global region.

For peer review only - http://bmjopen.bmj.com/site/about/guidelines.xhtml

Statistical methods

We described the participants by age group, sex, country of origin, prevalence of long-term conditions and EQ-5D-3L characteristics.

We used direct standardisation to calculate the age- and sex-adjusted prevalence of each disease and EQ-5D-3L problem by deprivation group, with the homeless group as the reference population. Standard errors were calculated using the method described by the Centers for Disease Control and Prevention.[13] We also calculated standardised prevalence ratios (SPRs) for each long-term condition and EQ-5D-3L problem, using the least deprived housed group as the reference population. Standard errors were √(SPR / expected number of cases).[14] To test for social gradients in the housed population, we reported p-values from logistic regression models with each disease and EQ-5D-3L problem as dependent variable and neighbourhood deprivation as a continuous independent variable, adjusting for age group and sex. We stratified prevalence of long-term conditions by age group among men (because there were insufficient cases among women) and tested the association between age and long-term conditions using chi-square tests. As sub-analyses, we calculated the age- and sex-adjusted prevalence of EQ-5D-3L problems by deprivation group among participants reporting any long-term condition; and analysed associations between multimorbidity, age and deprivation (results in appendix).

Six participants in the homeless group did not provide their sex and were excluded. Age and long-term condition data were complete. 142/1336 (10.6%) homeless participants and 3477/36673 (9.5%) housed participants did not complete all EQ-5D-3L questions, with similar age (chi-square p = 0.98) and sex (chi-square p = 0.60) characteristics to those with complete data. These participants were excluded from analysis of EQ-5D-3L but retained for other analyses.

All analyses were carried out using R version 3.4.2.

Patient involvement

Clients of the Find and Treat service were involved in selecting the diseases included in this study and in testing and refining the questionnaire.

RESULTS

Participants

The homeless sample consisted of 904 participants recruited from Birmingham and 432 from London (total = 1336). The response rate was 77% in London and 71% in Birmingham. The housed comparison group included 36673 participants. Assuming a uniform distribution of ages within each age category, the mean age of the homeless group was 39 and the housed group was 41. Eighty-one per cent (1087/1336) of the homeless group and 44% (16267/38009) of the housed group were male. A larger proportion of the homeless group was born outside of the UK. Table 1 summarises the characteristics of the study population.

For peer review only - http://bmjopen.bmj.com/site/about/guidelines.xhtml

<< Table 1 about here (see end of manuscript) >>

Long-term conditions

The adjusted prevalence of any long-term condition was 34.2% (95% CI 31.7%, 36.7%) in the homeless group and 11.7% (95% CI 11.3%, 12.2%) in the housed group. Asthma was the most commonly reported condition in both groups. All conditions were more common in the homeless group than in the housed group. COPD, heart problems and diabetes were strongly associated with older age for both homeless and housed groups, while epilepsy and asthma were not (figure 1 and online appendix, table). There were too few cases of stroke to observe an association with age.

<< figure 1 about here: Figure 1: Percentage of men that report long-term conditions, by age group >>

When we compared long-term conditions across deprivation quintiles in the housed population, we observed social gradients all diseases (p<0.0001), with participants living in deprived areas having higher risk (figure 2). For all conditions except diabetes, there were larger relative and absolute differences between the homeless group and all housed deprivation groups than there were between the housed deprivation groups. For example, the SPR comparing the risk of COPD in the most deprived quintile of housed participants versus the least deprived was 3.1 (95% CI 2.7, 3.1), compared to 17.2 (95% CI 14.7, 19.6) comparing the homeless group to the least deprived quintile.

<< figure 2 about here: Figure 2: Adjusted prevalence of long-term conditions (top panel) and standardised prevalence ratios (bottom panel), with 95% confidence intervals >>

Health-related quality of life (EQ-5D-3L)

The homeless group reported worse overall health, with a mean of 62.9 (95% CI 61.6, 64.2) on the EQ-5D-3L visual analogue scale, compared to an adjusted mean of 78.5 (95% CI 78.2, 78.9) for the housed group. Homeless men and women in all age groups reported worse health on the visual analogue scale (figure 3).

<< figure 3 about here: Figure 3: Mean EQ5D-Visual Analogue Scale score, by age, sex and homeless/housed status, with 95% confidence intervals. The values for 'all' show age- and sex-adjusted means >>

802/1194 (67%) of the homeless group reported problems in at least one of the EQ-5D-3L domains (mobility, self-care, usual activities, pain and anxiety), compared to 13239/33196 (40%) of the housed group. The homeless group reported more problems than the housed group in all five domains.

When we compared EQ-5D-3L problems across deprivation quintiles in the housed population, we observed social gradients for all domains (p<0.0001), with participants living in deprived areas more likely to report problems (figure 4). In contrast to the results for disease prevalence, the homeless group and the most deprived housed

For peer review only - http://bmjopen.bmj.com/site/about/guidelines.xhtml

quintile had similar likelihood of reporting problems related to mobility, self-care, usual activities and pain. For example, the SPR comparing the risk of problems with usual activities in the most deprived quintile of housed participants versus the least deprived was 2.6 (95% CI 2.4, 2.7) compared to 2.3 (95% CI 2.0, 2.7) for the homeless group. However, the homeless group had substantially greater likelihood of reporting problems with anxiety than all housed groups. The SPR comparing the risk of problems with anxiety in the most deprived quintile of housed participants versus the least deprived was 1.8 (95% CI 1.7, 1.8) compared to 4.0 (95% CI 3.7, 4.3) for the homeless group.

<< figure 4 about here: Figure 4: Health-related quality of life: adjusted prevalence of problems by deprivation group (top panel) and standardised prevalence ratios (bottom panel), with 95% confidence intervals >>

DISCUSSION

Principle findings

Homeless people report substantially worse health than those in stable housing and are three times more likely to report a chronic disease. In particular, homeless people report higher prevalence of asthma, COPD, epilepsy and heart problems. Most cases of long-term conditions studied here (apart from diabetes) can be attributed to homelessness and the circumstances associated with it. Homeless people also report worse quality of life, and are more than twice as likely to report problems with anxiety. When compared to the 'slopes' in health outcomes across deprivation quintiles, the inequalities in outcomes for homeless people appear more like a 'cliff'.

Limitations

This is a cross-sectional study and therefore offers limited insight into causation. The causal paths between homelessness and poor health are complex. Previous work has suggested that common precursors of homelessness include family problems in childhood, alcohol and illicit drugs, mental health problems and prison.[15] Physical health problems appear to follow homelessness. We did not attempt to control for lifestyle factors such as smoking, alcohol and illicit drugs for two reasons. First, they lie partially on the causal pathway between homelessness and disease (i.e. people may smoke, drink or use drugs because of homelessness), and second, we aimed to describe the prevalence rather than understand the causes of diseases.

The cross-sectional design also means that people who have already died are excluded. Given the age profile of the study population, this will be a small proportion of the housed group, with the UK Office for National Statistic's most recent life table suggesting that 88% of men and 91% of women survive to age 65.[16] However, cohort studies of homeless people suggest high rates of death at an early age. For example, 61 out of 273 (22%) hostel residents in Copenhagen aged 15-34 at baseline died during average follow-up of nine years.[17] Early death in

For peer review only - http://bmjopen.bmj.com/site/about/guidelines.xhtml

homeless groups is likely to mean that the least healthy homeless people are excluded from this study and the associations between homelessness and poor health are understated.

EQ-5D-3L has not been validated in homeless people. However, it has been used in previous studies of the health of homeless people[18] and validity has been demonstrated in overlapping groups such as people who use drugs.[19]

Comparisons with existing studies

The health of homeless people differs between countries and regions. However, the large inequalities between homeless and housed people are likely to persist. Studies that applied EQ-5D-3L to 73 homeless people in Italy[20] and 155 in Sweden[18] both found substantially more problems related to anxiety than other domains, reflecting our results and supporting similar health experiences between homeless people across high income countries. While there are few studies of long-term conditions in homeless people, existing studies have found similar results. For example, a study comparing risk of hospital admission between homeless people and the general population in Sweden found that homeless people had higher risk of hospitalisation in all disease groups studied.[8]

Implications

There are many possible causes of the 'cliff' in long-term conditions and anxiety observed for homeless people. Consumption of tobacco, alcohol and illicit drugs are more common among homeless people than the housed population.[4] Homelessness is associated with unmet needs for physical and mental healthcare,[21] meaning that conditions are often untreated.

Despite the higher prevalence of long-term conditions, homeless people did not report substantially more quality-of-life problems (apart from problems related to anxiety). When we looked at prevalence of EQ-5D-3L problems among those with any long-term condition (online appendix, figure), homeless participants were less likely to report problems than the most deprived participants in the housed group. This may reflect resilience and ability to cope with disease despite the adversity of homelessness.

The age of onset of long-term conditions is related to the patterns observed across age groups (figure 1). Asthma and epilepsy typically onset in childhood and the prevalence of these diseases is flat across age groups in both the housed and homeless populations. The reason for higher prevalence in the homeless group may relate to circumstances in childhood or before homelessness. Heavy alcohol use and brain injury[22] are common precursors of homelessness and may contribute to higher prevalence of epilepsy. COPD and heart problems are heavily associated with adult risk factors, particularly smoking, which reflects the strong association between these diseases and older age, and the higher prevalence in the homeless group. Similarly, most cases of diabetes can be attributed to adult risk factors and onset is typically from middle age. However, we did not observe a 'cliff' in the prevalence of diabetes

For peer review only - http://bmjopen.bmj.com/site/about/guidelines.xhtml

For peer review only

in homeless people. Key risk factors for diabetes, including obesity, physical inactivity and a sugary diet, may not differ between the homeless and housed groups. The results may also reflect differences in diagnosis. The average age of first stroke is older than the study population, at 71 (sd. 13) in men in the UK,[23] reflecting the small number of cases and limited power to observe inequalities, though higher prevalence was still observed in the homeless group (table 1).

Research into the health of homeless people has focused on infections and mental health. Evaluations of interventions have also focused on these areas. For example, there is evidence that case management can improve the effectiveness of treatments for mental health problems, drug dependence and tuberculosis in homeless people.[24] Research into 'housing first', an approach to healthcare that provides housing before medical treatment, also looks at how it can improve outcomes in these areas.[25]

The focus on infectious diseases and mental health conditions may have resulted from the high relative risks associated with homelessness.[26] However, mortality data suggest that cardiovascular diseases and cancers are the most common causes of death in homeless people despite lower relative risks, and are increasingly important among older homeless people.[26,27] The present study adds to this data by showing that respiratory diseases, epilepsy and heart problems are common among homeless people in the UK. Homeless people encounter barriers to management of chronic diseases[28] and there is a need for research into effective healthcare. Case management should be further explored for management of chronic diseases. Existing evidence-based preventative interventions should also be adapted and evaluated for excluded groups. For example, stop smoking interventions can reduce the risk of COPD and improve asthma symptoms, and should be tailored to homeless people and evaluated. There is also a need for routine surveillance data on the health of homeless people, to improve transparency and accountability for poor health in this population.

Homelessness is associated with childhood poverty[29] and early life adversity such as childhood abuse, neglect and being thrown out of home.[15,30] Adults in lower socioeconomic groups are more at risk of homelessness and the negative health impacts accumulate during homelessness. Research into disease management may improve outcomes for those with established disease, but addressing the 'cliff' in health experienced by homeless people will require a more sustained and comprehensive approach that reaches beyond the health sector.

For peer review only

For peer review only - http://bmjopen.bmj.com/site/about/guidelines.xhtml

TABLE

Table 1: characteristics of study population

		**Find & Treat (homeless)**	**Health Survey for England (housed)**
Sample size		1336	36673
Age group	16-24	10.5 (140)	13.7 (5028)
% (n)	25-34	27.2 (364)	18.9 (6945)
	35-44	28.0 (374)	23.1 (8486)
	45-54	23.6 (315)	22.8 (8378)
	55-64	10.7 (143)	21.4 (7836)
Sex	Female	18.6 (249)	55.6 (20406)
% (n)	Male	81.4 (1087)	44.4 (16267)
Country of birth*	UK	65.1 (870)	71.3
% (n)	Africa	8.0 (107)	5.8
	Eastern Europe	10.7 (143)	4.1
	South Asia	2.1 (28)	6.8
	Other	10.2 (136)	12.1
	Unknown	3.9 (52)	-
Prevalence of long-term conditions	Asthma	18.3 (244)	5.8 (2138)
	COPD	14 (187)	1.6 (573)
% (n)	Epilepsy	6.0 (80)	0.8 (304)
	Heart problems	7.7 (103)	2.4 (870)
	Stroke	1.8 (24)	0.4 (149)
	Diabetes	4.1 (55)	3.3 (1203)
**EQ-5D-3L (health-related quality of life)**			
Reporting problems**	Mobility	21 (251)	12.1 (4017)
	Self-care	7.7 (92)	3.7 (1228)
% (n)	Usual activities	14.9 (178)	12.7 (4226)
	Pain	36.2 (432)	28.9 (9584)
	Anxiety	54.1 (646)	21.0 (6960)
Visual analogue scale Mean (sd)		62.8 (22.4)	78.1 (17.4)

*Country of birth proportions for the comparison group are a proxy from the UK Census 2011 participants aged 16-64 living in London and the West Midlands (23,794,053 respondents)
**The proportions exclude 142 cases in the Find & Treat group and 3477 cases in the Health Survey for England group who did not answer all EQ-5D-3L questions

For peer review only - http://bmjopen.bmj.com/site/about/guidelines.xhtml

ADDITIONAL INFORMATION

Funding

Dr Robert W Aldridge is funded by a Wellcome Trust Clinical Research Career Development Fellowship (206602/Z/17/Z). Dr Imtiaz Ahmed is funded by Sandwell Hospital and Public Health England. Ms Serena A Luchenski is funded by National Institute of Health Research and Health Education England Clinical Doctoral Research Fellowship.

Contributions

AS and AH conceived of the initial idea. MD, AS, DM and CS contributed to data collection. All authors contributed to the design of the study. DL conducted data analysis and wrote the first draft of the paper. All authors contributed to the main content of the manuscript, provided comments on the final draft, and approved the manuscript before submission.

Competing interests

AH is Trustee of the UK-based charity 'Pathway (healthcare for homeless people)'. AS is Clinical Lead for the Find & Treat Service; data was collected from homeless clients of this service.

Data sharing statement

Data from the survey of homeless people has not been made publicly available because the some individuals may be identifiable. Data from the Health Survey for England used in this study is available via the UK Data Service, serial numbers SN6397, SN6986, SN7260, SN7480, SN7919.

For peer review only

REFERENCES

1 Mackenbach J, Kunst A, Cavelaars A, *et al.* Socioeconomic inequalities in morbidity and mortality in western Europe. *Lancet* 1997;**349**:1655–9. doi:10.1016/S0140-6736(96)07226-1

2 Marmot MG, Stansfeld S, Patel C, *et al.* Health inequalities among British civil servants: the Whitehall II study. *Lancet* 1991;**337**:1387–93. doi:10.1016/0140-6736(91)93068-K

3 Aldridge RW, Story A, Hwang SW, *et al.* Morbidity and mortality in homeless individuals, prisoners, sex workers, and individuals with substance use disorders in high-income countries: a systematic review and meta-analysis. *Lancet* 2017;**6736**:1–10. doi:10.1016/S0140-6736(17)31869-X

4 Homeless Link. The unhealthy state of homelessness: Health audit results. 2014.http://www.homeless.org.uk/sites/default/files/site-attachments/The unhealthy state of homelessness FINAL.pdf (accessed 27 Jun 2018).

5 Keogh C, O'Brien KK, Hoban A, *et al.* Health and use of health services of people who are homeless and at risk of homelessness who receive free primary health care in Dublin. *BMC Health Serv Res* 2015;**15**:58. doi:https://dx.doi.org/10.1186/s12913-015-0716-4

6 Brett T, Arnold-Reed DE, Troeung L, *et al.* Multimorbidity in a marginalised, street-health Australian population: a retrospective cohort study. *BMJ Open* 2014;**4**:e005461–e005461. doi:10.1136/bmjopen-2014-005461

7 Bines W. *The health of single homeless people*. York: : Centre for Housing Policy, University of York 1994.

8 Beijer U, Bruce D, Burström B. Changes over time in the risk of hospitalization for physical diseases among homeless men and women in Stockholm: A comparison of two cohorts. *Scand J Public Health* 2016;**44**:784–90. doi:10.1177/1403494816671601

9 Fazel S, Geddes JR, Kushel M. The health of homeless people in high-income countries: descriptive epidemiology, health consequences, and clinical and policy recommendations. *Lancet* 2014;**384**:1529–40. doi:10.1016/S0140-6736(14)61132-6

10 Craig R, Fuller E, Mindell J. Health Survey for England 2014, Volume 2: Methods and Documentation. 2014.https://files.digital.nhs.uk/publicationimport/pub19xxx/pub19295/hse2014-methods-and-docs.pdf (accessed 27 Jun 2018).

11 The EuroQol Group. EuroQol - a new facility for the measurement of health-related quality of life. *Health Policy (New York)* 1990;**16**:199–208. doi:10.1016/0168-8510(90)90421-9

12 DCLG. The English Indices of Deprivation 2010. Neighb. Stat. Release. 2011.https://assets.publishing.service.gov.uk/government/uploads/system/uploads/attachment_data/file/6320/1870718.pdf (accessed 27 Jun 2018).

13 Centers for Disease Control and Prevention. Age Standardization and Population Counts.

For peer review only - http://bmjopen.bmj.com/site/about/guidelines.xhtml

2014.https://www.cdc.gov/nchs/tutorials/NHANES/NHANESAnalyses/agestandardization/age_standardization_intro.htm (accessed 27 Jun 2018).

14 Armitage P, Berry G, Matthews JNS. Statistical Methods in Epidemiology. In: *Statistical Methods in Medical Research, Fourth Edition*. Massachusetts: : Blackwell Science 2008.

15 Fitzpatrick S, Bramley G, Johnsen S. Pathways into Multiple Exclusion Homelessness in Seven UK Cities. *Urban Stud* 2012;**50**:148–68. doi:10.1177/0042098012452329

16 Office of National Statistics. National life tables, UK: 2014 to 2016. 2017.https://www.ons.gov.uk/peoplepopulationandcommunity/birthsdeathsandmarriages/lifeexpectancies/bulletins/nationallifetablesunitedkingdom/2014to2016 (accessed 27 Jun 2018).

17 Nordentoft M, Wandall-Holm N. 10 year follow up study of mortality among users of hostels for homeless people in Copenhagen. *BMJ* 2003;**327**:81. doi:10.1136/bmj.327.7406.81

18 Sun S, Irestig R, Burström B, *et al.* Health-related quality of life (EQ-5D) among homeless persons compared to a general population sample in Stockholm County, 2006. *Scand J Soc Med* 2012;**40**:115–25. doi:10.1177/1403494811435493

19 Goranitis I, Coast J, Day E, *et al.* Measuring Health and Broader Well-Being Benefits in the Context of Opiate Dependence: The Psychometric Performance of the ICECAP-A and the EQ-5D-5L. *Value Heal* 2016;**19**:820–8. doi:10.1016/j.jval.2016.04.010

20 Levorato S, Bocci G, Troiano G, *et al.* Health status of homeless persons: a pilot study in the Padua municipal dorm. *Ann Ig* 2017;**29**:54–62. doi:https://dx.doi.org/10.7416/ai.2017.2132

21 Palepu A, Gadermann A, Hubley AM, *et al.* Substance Use and Access to Health Care and Addiction Treatment among Homeless and Vulnerably Housed Persons in Three Canadian Cities. *PLoS One* 2013;**8**. doi:10.1371/journal.pone.0075133

22 Topolovec-Vranic J, Ennis N, Colantonio A, *et al.* Traumatic brain injury among people who are homeless: A systematic review. *BMC Public Health* 2012;**12**. doi:10.1186/1471-2458-12-1059

23 Lee S, Shafe ACE, Cowie MR. UK stroke incidence, mortality and cardiovascular risk management 1999-2008: time-trend analysis from the General Practice Research Database. *BMJ Open* 2011;**1**:e000269–e000269. doi:10.1136/bmjopen-2011-000269

24 Hwang SW, Tolomiczenko G, Kouyoumdjian FG, *et al.* Interventions to Improve the Health of the Homeless: A Systmatic Review. *Am J Prev Med* 2005;**29**:311.e1-311.e9. doi:10.1016/j.amepre.2005.06.017

25 Woodhall-Melnik JR, Dunn JR. A systematic review of outcomes associated with participation in Housing First programs. *Hous Stud* 2016;**31**:287–304. doi:10.1080/02673037.2015.1080816

26 Fugelstad A, Beijer U, Andreasson S, *et al.* Mortality and causes of death

For peer review only - http://bmjopen.bmj.com/site/about/guidelines.xhtml

among homeless women and men in Stockholm. *Scand J Public Health* 2011;**39**:121–7. doi:10.1177/1403494810393554

27 Thomas B. Homelessness kills: An analysis of the mortality of homeless people in early twenty-first century England. 2012.https://www.crisis.org.uk/media/236798/crisis_homelessness_kills2012.pdf (accessed 27 Jun 2018).

28 Hwang SW, Bugeja AL. Barriers to appropriate diabetes management among homeless people in Toronto. *CMAJ* 2000;**163**:161–5.

29 Bramley G, Fitzpatrick S. Homelessness in the UK: who is most at risk ? *Hous Stud* 2018;**3037**:1–21. doi:10.1080/02673037.2017.1344957

30 Bramley G, Fitzpatrick S, Edwards J, *et al.* Hard Edges: Mapping Severe and Multiple Disadvantage. 2015.http://lankellychase.org.uk/wp-content/uploads/2015/07/Hard-Edges-Mapping-SMD-2015.pdf (accessed 27 Jun 2018).

For peer review only

For peer review only - http://bmjopen.bmj.com/site/about/guidelines.xhtml

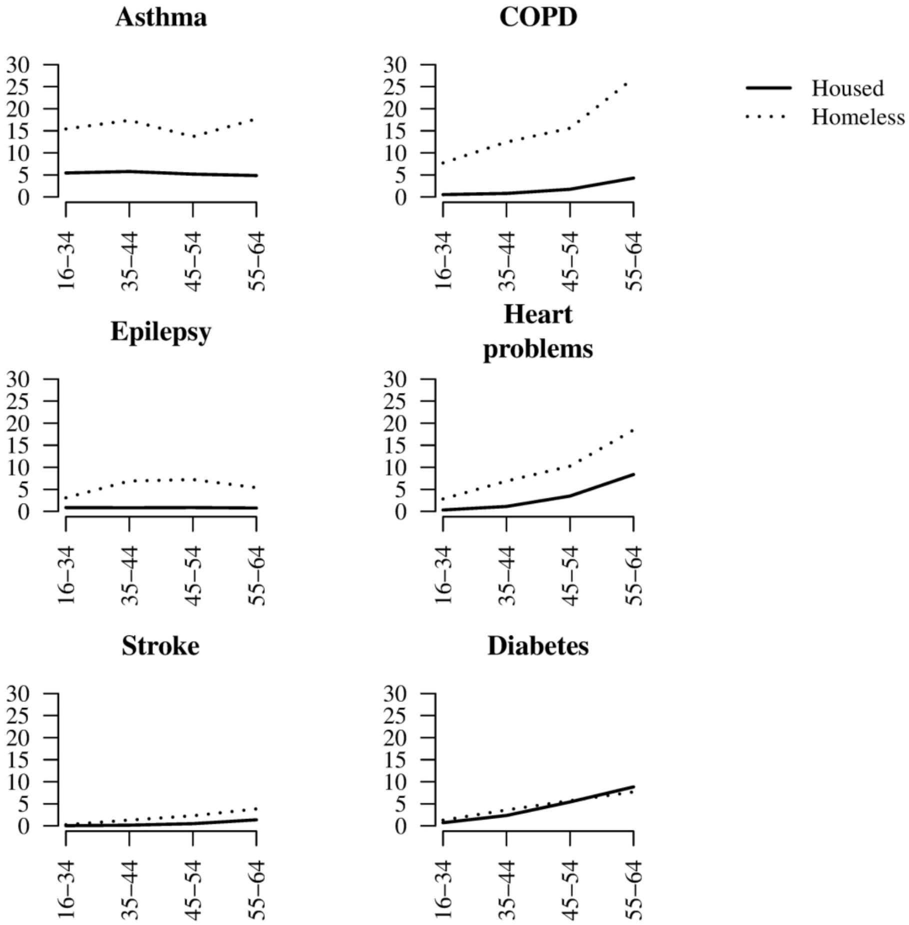

Figure 1: Percentage of men that report long-term conditions, by age group

177x177mm (300 x 300 DPI)

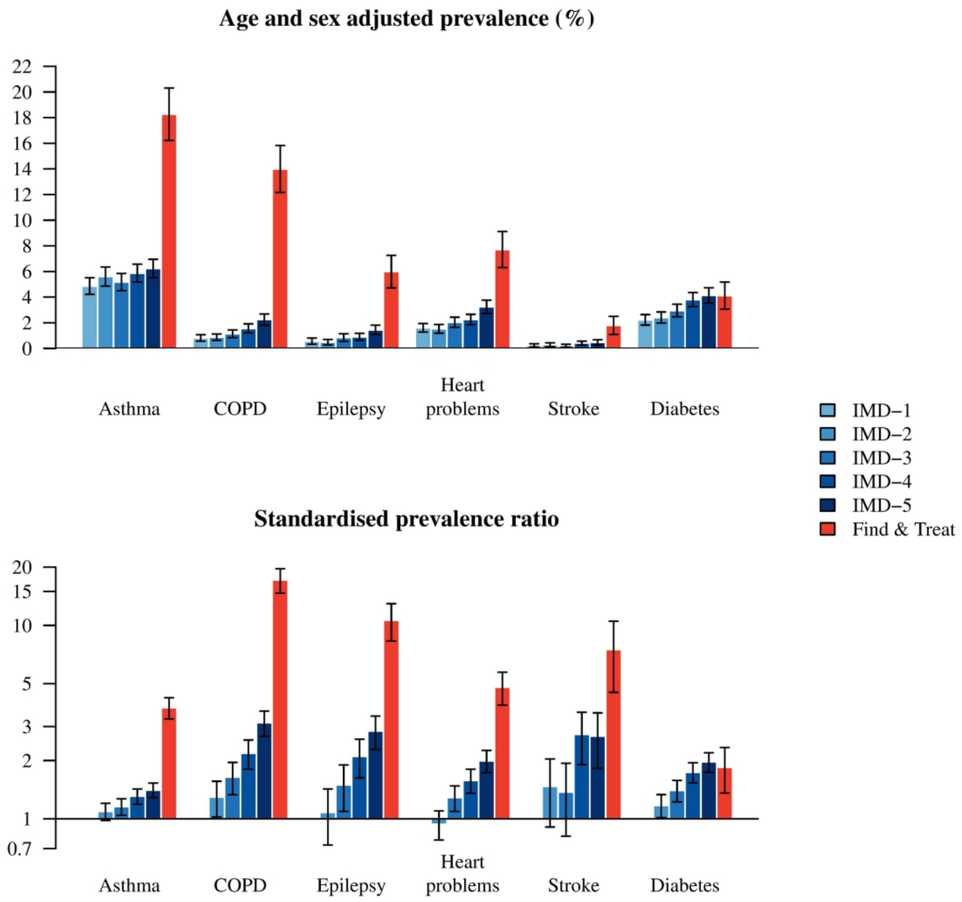

Figure 2: Adjusted prevalence of long-term conditions (top panel) and standardised prevalence ratios (bottom panel), with 95% confidence intervals

177x177mm (300 x 300 DPI)

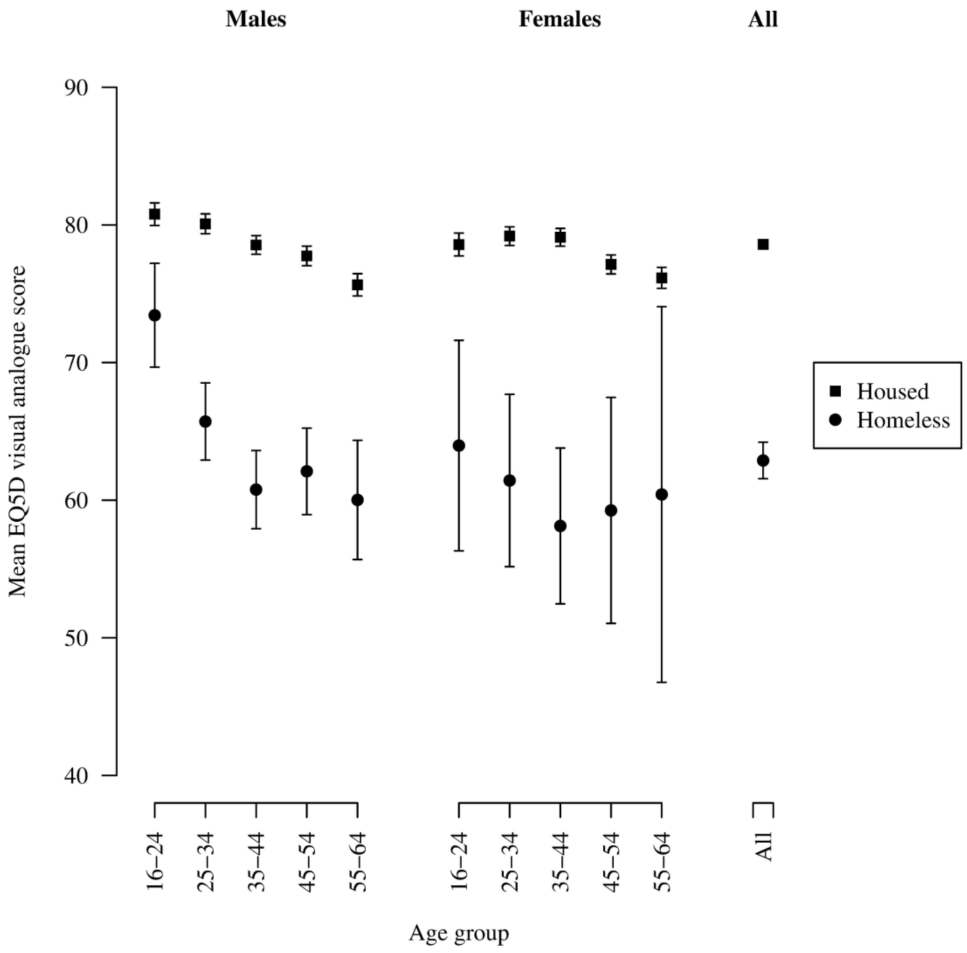

Figure 3: Mean EQ5D-Visual Analogue Scale score, by age, sex and homeless/housed status, with 95% confidence intervals. The values for 'all' show age- and sex-adjusted means

177x177mm (300 x 300 DPI)

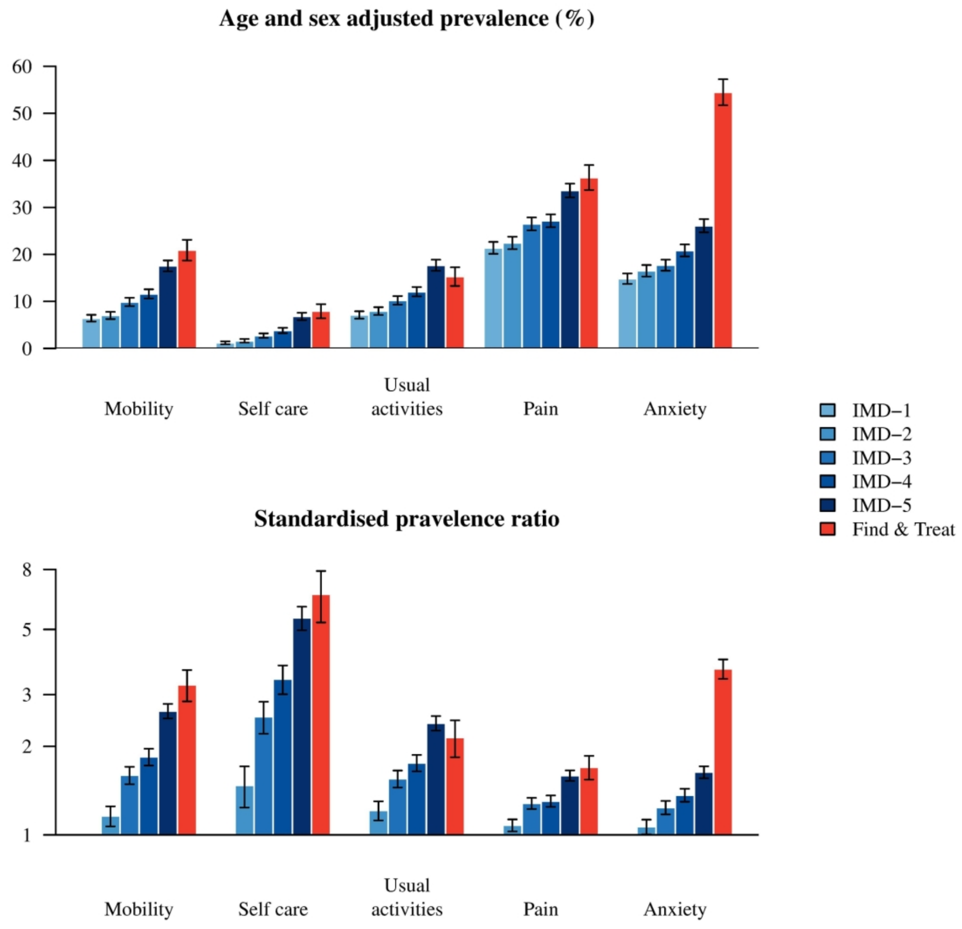

Figure 4: Health-related quality of life: adjusted prevalence of problems by deprivation group (top panel) and standardised prevalence ratios (bottom panel), with 95% confidence intervals

177x177mm (300 x 300 DPI)

Online appendix

1. Table: prevalence of long term conditions in men by age group
2. Figure. Health-related quality of life in participants with one or more long-term condition
3. Associations with multimorbidity

For peer review only

For peer review only - http://bmjopen.bmj.com/site/about/guidelines.xhtml

1. Table. prevalence of long-term condition in men by age group: cases/n (prevalence; 95% confidence intervals). Stroke is excluded due to small numbers of cases within age groups

Condition	Age group	Find & Treat (homeless)	Health Survey for England (housed)
Asthma	16-34	60/389 (15.4; 12.1-19.5)	283/5205 (5.4; 4.8-6.1)
	35-44	53/305 (17.4; 13.4-22.2)	216/3744 (5.8; 5.1-6.6)
	45-54	36/263 (13.7; 9.9-18.6)	190/3672 (5.2; 4.5-6.0)
	55-64	23/130 (17.7; 11.8-25.6)	177/3646 (4.9; 4.2-5.6)
		p = 0.61	p = 0.34
COPD	16-34	30/389 (7.7; 5.3-10.9)	28/5205 (0.5; 0.4-0.8)
	35-44	38/305 (12.5; 9.1-16.8)	30/3744 (0.8; 0.6-1.2)
	45-54	41/263 (15.6; 11.5-20.7)	64/3672 (1.7; 1.4-2.2)
	55-64	35/130 (26.9; 19.7-35.5)	156/3646 (4.3; 3.7-5.0)
		p < 0.001	p < 0.001
Epilepsy	16-34	12/389 (3.1; 1.7-5.5)	45/5205 (0.9; 0.6-1.2)
	35-44	21/305 (6.9; 4.4-10.5)	31/3744 (0.8; 0.6-1.2)
	45-54	19/263 (7.2; 4.5-11.2)	32/3672 (0.9; 0.6-1.2)
	55-64	7/130 (5.4; 2.4-11.2)	28/3646 (0.8; 0.5-1.1)
		p = 0.07	p = 0.96
Heart problems	16-34	11/389 (2.8; 1.5-5.2)	17/5205 (0.3; 0.2-0.5)
	35-44	21/305 (6.9; 4.4-10.5)	42/3744 (1.1; 0.8-1.5)
	45-54	27/263 (10.3; 7.0-14.7)	128/3672 (3.5; 2.9-4.1)
	55-64	24/130 (18.5; 12.4-26.4)	305/3646 (8.4; 7.5-9.3)
		p < 0.001	p < 0.001
Diabetes	16-34	5/389 (1.3; 0.5-3.1)	37/5205 (0.7; 0.5-1.0)
	35-44	11/305 (3.6; 1.9-6.5)	88/3744 (2.4; 1.9-2.9)
	45-54	15/263 (5.7; 3.3-9.4)	199/3672 (5.4; 4.7-6.2)
	55-64	10/130 (7.7; 4.0-14.1)	322/3646 (8.8; 7.9- 9.8)
		p = 0.002	p < 0.001

Age groups 16-24 and 25-34 have been merged to avoid publication of small numbers. p-values are based on chi-square tests.

For peer review only - http://bmjopen.bmj.com/site/about/guidelines.xhtml

2. Figure. Health-related quality of life in participants with one or more long-term condition: adjusted prevalence of any problems by deprivation group (top panel) and standardised prevalence ratios (bottom panel), with 95% confidence intervals

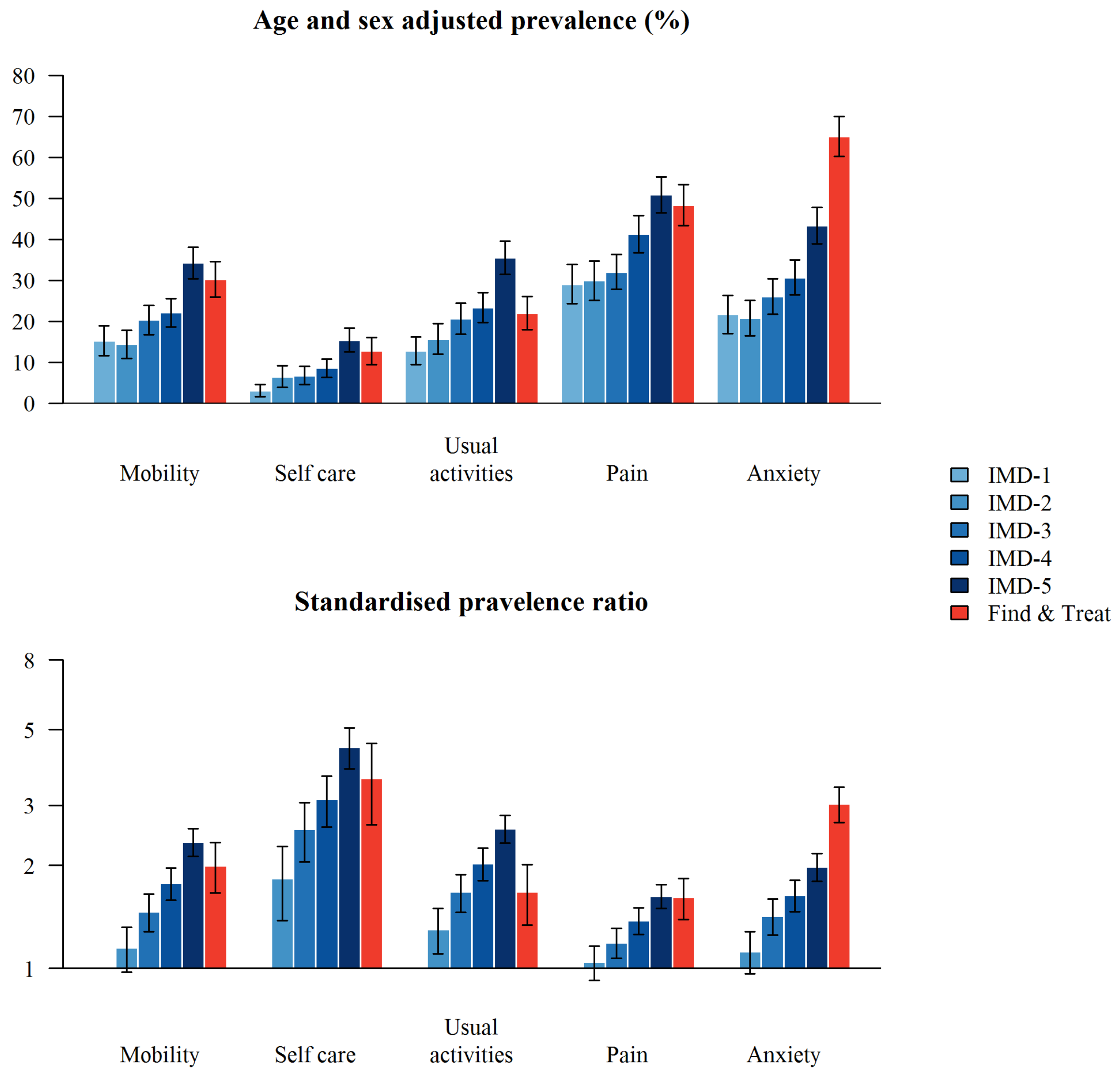

For peer review only - http://bmjopen.bmj.com/site/about/guidelines.xhtml

3. Associations with multimorbidity

For the purposes of multimorbidity analysis, we combined chronic respiratory diseases (asthma and chronic obstructive pulmonary disease). Multimorbidity was then defined as report of two or more diseases.

(a) Table: prevalence of multimorbidity in men by age group: cases/n (prevalence; 95% confidence intervals). Women are excluded to avoid publication of small numbers.

Age group	Find & Treat (homeless)	Health Survey for England (housed)
16-34	11/389 (2.8; 1.5-5.2)	11/5205 (0.2; 0.1-0.4)
35-44	19/305 (6.2; 3.9- 9.7)	25/3744 (0.7; 0.4-1.0)
45-54	24/263 (9.1; 6.1-13.4)	68/3672 (1.9; 1.5-2.4)
55-64	20/130 (15.4; 9.9-23.0)	123/3646 (3.4; 2.8-4.0)
	$p < 0.0001$	$p < 0.0001$

Age groups 16-24 and 25-34 have been merged to avoid publication of small numbers. p-values are based on chi-square tests.

(b) Figure: age- and sex-adjusted prevalence of multimorbidity by deprivation group (left panel) and standardised prevalence ratio (right panel), with 95% confidence intervals

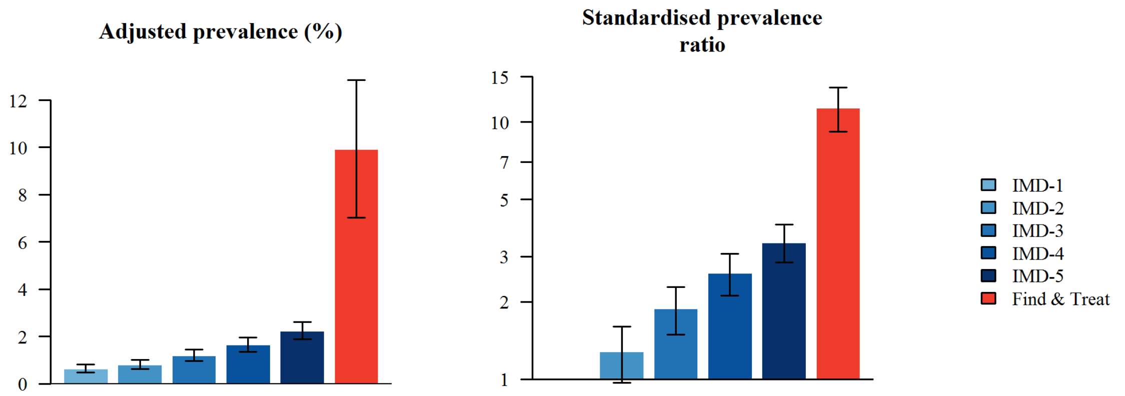

For peer review only - http://bmjopen.bmj.com/site/about/guidelines.xhtml

STROBE Statement—Checklist of items that should be included in reports of ***cross-sectional studies***

	Item No	Recommendation		Page number
**Title and abstract**	1	(*a*) Indicate the study's design with a commonly used term in the title or the abstract	Yes	1
		(*b*) Provide in the abstract an informative and balanced summary of what was done and what was found	Yes	2
**Introduction**				
Background/rationale	2	Explain the scientific background and rationale for the investigation being reported	Yes	3
Objectives	3	State specific objectives, including any prespecified hypotheses	Yes	3
**Methods**				
Study design	4	Present key elements of study design early in the paper	Yes	2-5
Setting	5	Describe the setting, locations, and relevant dates, including periods of recruitment, exposure, follow-up, and data collection	Yes	3-4
Participants	6	(*a*) Give the eligibility criteria, and the sources and methods of selection of participants	Yes	3-4
Variables	7	Clearly define all outcomes, exposures, predictors, potential confounders, and effect modifiers. Give diagnostic criteria, if applicable	Yes	4
Data sources/ measurement	8*	For each variable of interest, give sources of data and details of methods of assessment (measurement). Describe comparability of assessment methods if there is more than one group	Yes	4-5
Bias	9	Describe any efforts to address potential sources of bias	Yes	5
Study size	10	Explain how the study size was arrived at	N/A	
Quantitative variables	11	Explain how quantitative variables were handled in the analyses. If applicable, describe which groupings were chosen and why	Yes	4-5
Statistical methods	12	(*a*) Describe all statistical methods, including those used to control for confounding	Yes	5
		(*b*) Describe any methods used to examine subgroups and interactions	Yes	5
		(*c*) Explain how missing data were addressed	Yes	5
		(*d*) If applicable, describe analytical methods taking account of sampling strategy	N/A	
		(*e*) Describe any sensitivity analyses	N/A	
**Results**				
Participants	13*	(a) Report numbers of individuals at each stage of study—eg numbers potentially eligible, examined for eligibility, confirmed eligible, included in the study, completing follow-up, and analysed	Yes	5
		(b) Give reasons for non-participation at each stage	N/A	
		(c) Consider use of a flow diagram	N/A	
Descriptive data	14*	(a) Give characteristics of study participants (eg	Yes	5, 10

For peer review only

For peer review only - http://bmjopen.bmj.com/site/about/guidelines.xhtml

		demographic, clinical, social) and information on exposures and potential confounders		
		(b) Indicate number of participants with missing data for each variable of interest	Yes	5
Outcome data	15*	Report numbers of outcome events or summary measures	Yes	5-6
Main results	16	(*a*) Give unadjusted estimates and, if applicable, confounder-adjusted estimates and their precision (eg, 95% confidence interval). Make clear which confounders were adjusted for and why they were included	Yes	10, 6-7
		(*b*) Report category boundaries when continuous variables were categorized	Yes	4 (age)
		(*c*) If relevant, consider translating estimates of relative risk into absolute risk for a meaningful time period	N/A	
Other analyses	17	Report other analyses done—eg analyses of subgroups and interactions, and sensitivity analyses	Yes	Appendix
**Discussion**				
Key results	18	Summarise key results with reference to study objectives	Yes	7
Limitations	19	Discuss limitations of the study, taking into account sources of potential bias or imprecision. Discuss both direction and magnitude of any potential bias	Yes	7-8
Interpretation	20	Give a cautious overall interpretation of results considering objectives, limitations, multiplicity of analyses, results from similar studies, and other relevant evidence	Yes	8-9
Generalisability	21	Discuss the generalisability (external validity) of the study results	Yes	8
**Other information**				
Funding	22	Give the source of funding and the role of the funders for the present study and, if applicable, for the original study on which the present article is based	Yes	11

*Give information separately for exposed and unexposed groups.

Note: An Explanation and Elaboration article discusses each checklist item and gives methodological background and published examples of transparent reporting. The STROBE checklist is best used in conjunction with this article (freely available on the Web sites of PLoS Medicine at http://www.plosmedicine.org/, Annals of Internal Medicine at http://www.annals.org/, and Epidemiology at http://www.epidem.com/). Information on the STROBE Initiative is available at www.strobe-statement.org.

For peer review only

BMJ Open

Health-related quality of life and prevalence of six chronic diseases in homeless and housed people: a cross-sectional study in London and Birmingham, England

Journal:	*BMJ Open*
Manuscript ID	bmjopen-2018-025192.R1
Article Type:	Research
Date Submitted by the Author:	14-Jan-2019
Complete List of Authors:	Lewer, Dan; University College London, Institute of Epidemiology and Healthcare<br>Aldridge, Robert; University College London, Farr Institute of Health Informatics Research<br>Menezes, Dee; University College London, UCL Farr Institute of Health Informatics Research<br>Sawyer, Clare; University College London Hospitals NHS Foundation Trust, Find & Treat<br>Zaninotto, Paola; University College London,<br>Dedicoat, Martin; Heart of England NHS Foundation Trust<br>Ahmed, Imtiaz; Sandwell and West Birmingham Hospitals NHS Trust<br>Luchenski, Serena; University College London, UCL Farr Institute of Health Informatics Research<br>Hayward, Andrew; University College London, Infection and Population Health<br>Story, Alistair; University College London Hospitals NHS Foundation Trust, Find & Treat
**Primary Subject Heading**:	Public health
Secondary Subject Heading:	Cardiovascular medicine, Diabetes and endocrinology, Epidemiology, Neurology, Respiratory medicine
Keywords:	PUBLIC HEALTH, EPIDEMIOLOGY, Homelessness, Inequality

SCHOLARONE™
Manuscripts

Health-related quality of life and prevalence of six chronic diseases in homeless and housed people: a cross-sectional study in London and Birmingham, England

Authors

Dan Lewer (MSc)[1,2], Robert W Aldridge (PhD)[1], Dee Menezes (MPH) [1], Clare Sawyer (MSc)[3], Paola Zaninotto (PhD) [2], Martin J Dedicoat (PhD)[4], Imtiaz Ahmed (FRCP)[5], Serena Luchenski (FFPH)[1], Andrew Hayward (MD)[2]*, Alistair Story (PhD)[2,3]*

*Joint senior authors

Affiliations

1. Institute of Health Informatics, University College London, 222 Euston Road, London NW1 2DA, United Kingdom
2. Institute of Epidemiology and Healthcare, University College London, 1-19 Torrington Place, London WC1E 6BT, United Kingdom
3. Find and Treat, University College London Hospitals NHS Foundation Trust, 250 Euston Road, London NW1 2PG, United Kingdom
4. Heart of England NHS Foundation Trust, Bordesley Green East, Birmingham B9 5SS, United Kingdom
5. Sandwell and West Birmingham Hospitals NHS Trust, Sandwell General Hospital, West Bromwich B71 4HJ, United Kingdom

Corresponding author

Dan Lewer, d.lewer@ucl.ac.uk, +44 (0) 203 549 5142

Word count: 2,717

For peer review only

ABSTRACT

Objectives: To compare health-related quality of life and prevalence of chronic diseases in housed and homeless populations.

Design: Cross-sectional survey with an age- and sex-matched housed comparison group.

Setting: Hostels, day centres and soup runs in London and Birmingham, England.

Participants: Homeless participants were either sleeping rough or living in hostels and had a history of sleeping rough. The comparison group was drawn from the Health Survey for England. The study included 1336 homeless and 13360 housed participants.

Outcome measures: Chronic diseases were self-reported asthma, chronic obstructive pulmonary disease, epilepsy, heart problems, stroke, and diabetes. Health-related quality of life was measured using EQ-5D-3L.

Results: Housed participants in more deprived neighbourhoods were more likely to report disease. Homeless participants were substantially more likely than housed participants in the most deprived quintile to report all diseases except diabetes (which had similar prevalence in homeless participants and the most deprived housed group). For example, the prevalence of chronic obstructive pulmonary disease was 1.1% (95% CI 0.7%, 1.6%) in the least deprived housed quintile; 2.0% (95% CI 1.5%, 2.6%) in the most deprived housed quintile; and 14.0% (95% CI 12.2%, 16.0%) in the homeless group. Social gradients were also seen for problems in each EQ-5D-3L domain in the housed population, but homeless participants had similar likelihood of reporting problems as the most deprived housed group. The exception was problems related to anxiety, which were substantially more common in homeless people than any of the housed groups.

Conclusions: While differences in health between housed socioeconomic groups can be described as a ‘slope’, differences in health between housed and homeless people are better understood as a ‘cliff’.

For peer review only - http://bmjopen.bmj.com/site/about/guidelines.xhtml

STRENGTHS AND LIMITATIONS OF THIS STUDY

- The study is based on a large survey of 1,336 homeless people.
- The survey includes data on outcomes that are rarely measured in homeless people, including long-term health conditions and health-related quality of life.
- An age- and sex-matched comparison group is used to compare the results with the general population.
- The cross sectional design means that causal pathways between homelessness and health are not investigated.
- The data was collected as part of a service evaluation and does not include comprehensive data across health problems and risk factors.

For peer review only

For peer review only - http://bmjopen.bmj.com/site/about/guidelines.xhtml

INTRODUCTION

Homelessness is an enduring social problem in high income countries [1,2]. It is associated with poor health outcomes. Cohort studies have shown that mortality risk for homeless people is three to six times the general population [3–7]. The most common causes of death are usually (but not always [7]) accidents, drug overdoses, suicides and liver diseases. Homeless people also have substantially increased risk of death due to cardiovascular and respiratory diseases [3,5,7,8].

There are few studies of morbidity among homeless people. Most have examined infections and mental health problems [9,10]; finding high relative and absolute frequencies. The few studies looking at broader outcomes have found that respiratory diseases, dental problems, headaches and skin diseases are also more common among homeless people than the general population [11–15].

Inequalities in health in the general population have been widely observed. People in lower socioeconomic groups have more risk factors and worse health outcomes [16,17]. Although poor health has been observed in homeless people, it is unclear whether homeless people have substantially worse health than the most deprived groups in the general population. We analysed data from a large survey of homeless people to understand how health-related quality of life and prevalence of chronic diseases in homeless people compares to different socioeconomic groups in the general population.

For peer review only - http://bmjopen.bmj.com/site/about/guidelines.xhtml

METHODS

Data sources

We used anonymised data from an evaluation of a mobile tuberculosis screening unit (‘Find and Treat’) that works alongside services for homeless people. Participants were people sleeping rough and accessing day centres and soup kitchens, or people with a history of rough sleeping who are living in temporary hostel accommodations. Staff at the screening unit worked with homeless people to develop a health survey including questions on chronic diseases and health-related quality of life. Interviewer-led questionnaires were conducted in London from 1 July 2012 to 31 August 2012 and in Birmingham from 13 July 2014 to 18 July 2014 and from 16 March 2015 to 24 July 2015. We excluded 21 participants aged under 16 or over 64.

We selected a housed comparison group from the Health Survey for England. This is an annual series of cross-sectional surveys designed to be representative of adults living in private households. Participants are selected based on stratified random sampling of households across England. The full method is described elsewhere [18]. We used data from the 2006, 2008, 2010, 2011, 2012 and 2014 surveys, limiting to participants from urban areas. Within each deprivation quintile, we stratified participants by age group and sex, and within each stratum selected a random sample of size double the number of homeless participants in the corresponding age and sex group. This produced an age- and sex-matched comparison group with ten housed participants for each homeless participant.

Variables

The main outcomes are self-reported long-term conditions and health-related quality of life.

To establish prevalence of long-term conditions, homeless participants were asked whether a doctor or nurse had ever given a diagnosis of (i) asthma, (ii) bronchitis, emphysema or obstructive airways disease (abbreviated to COPD, chronic obstructive pulmonary disease), (iii) epilepsy, (iv) heart problems, including heart attack, angina, murmur or abnormal heart rhythms, (v) stroke, and (vi) diabetes. Housed participants were asked whether they had a health condition lasting or expected to last 12 months or more, with corresponding options of (i) asthma, (ii) bronchitis, emphysema or other respiratory complaints, (iii) epilepsy, fits or convulsions, (iv) heart attack, angina or other heart problems, (v) stroke, cerebral haemorrhage or cerebral thrombosis and (vi) diabetes including hyperglycaemia.

EQ-5D-3L [19] was used to measure health-related quality of life. Participants report no problems, some problems or extreme problems in five domains: mobility, pain, self-care, usual activities and anxiety. We created a binary variable showing whether participants reported ‘none’ vs. ‘some’ or ‘extreme’ problems’. Participants also completed a visual-analogue scale (VAS) of overall health on the day of interview, where 0 is the worst imaginable state and 100 the best.

For peer review only - http://bmjopen.bmj.com/site/about/guidelines.xhtml

The main exposure was deprivation. The homeless group were classified as the most deprived, while the housed comparison group were classified using quintiles of the Index of Multiple Deprivation 2007.[20] This is an area-based index derived from levels of income, employment, health, disability, education, skills and training in small local areas of roughly 1500 residents.

Age was grouped into 16-24, 25-34, 35-44, 45-54 and 55-64, which was consistently available across datasets.

We also reported health behaviours: current smoking, drinking every day in the past week, and currently injecting drugs.

Statistical methods

We described homeless and housed participants by age group, sex, health behaviours, prevalence of long-term conditions and EQ-5D-3L characteristics. Chi-squared tests were used to test the difference between categorical variables and a Wilcoxon rank-sum test was used to test the difference between the EQ-5D-3L VAS score. We additionally calculated the prevalence of long-term conditions and EQ-5D-3L problems by deprivation group and calculated prevalence ratios with the least deprived housed group as the reference population, with Wald confidence intervals. To test for social gradients in the housed population, we used chi-square test for trends with deprivation quintiles scored 1-5. We stratified prevalence of long-term conditions by age group and tested the association between age and long-term conditions using chi-square tests.

As a sub-analysis of the effect of morbidity on inequalities in health-related quality of life, we used logistic regression to estimate the joint effect of deprivation and any morbidity (with the least deprived group with no long-term conditions as the reference group) on each EQ-5D-3L domain, adjusting for age group and sex. The results are shown in the Supplementary Information.

Some of the Health Survey for England data were collected several years before the surveys of homeless people, so we conducted a sensitivity analysis in which the procedures described above were repeated without the Health Survey for England years 2006 and 2008. These results are shown in Supplementary Information.

Six participants in the homeless group did not provide their sex and were excluded. Age and long-term condition data were complete. 142/1336 (10.6%) homeless participants and 1353/13360 (10.1%) housed participants did not complete all EQ-5D-3L questions, with similar age (chi-square $p = 0.40$) and sex (chi-square $p = 0.61$) characteristics to those with complete data. These participants were excluded from analysis of EQ-5D-3L but retained for other analyses. Data on current smoking and everyday drinking was missing for 904/1336 (68%) homeless participants because these questions were only asked to participants in London. These data were missing for less than 1% of the housed group (table 1). Drug injection data was missing for 29/1336 (2.2%) homeless participants and all housed participants (as questions about drug injection are not included in the Health Survey for England). Data on health behaviours are shown in Table 1 but associations with outcomes were not analysed.

For peer review only - http://bmjopen.bmj.com/site/about/guidelines.xhtml

All analyses were carried out using R version 3.4.2.

Patient involvement

Clients of the Find and Treat service were involved in selecting the diseases included in this study, and in testing and refining the questionnaire.

Ethics

This was an analysis of anonymised secondary data. We checked the requirement for ethical approval for this analysis using the NHS Health Research Authority / MRC approval checklist [21] and determined that ethical approval or additional informed consent was not required.

For peer review only

For peer review only - http://bmjopen.bmj.com/site/about/guidelines.xhtml

RESULTS

Participants

The homeless sample consisted of 904 participants recruited from Birmingham and 432 from London (total = 1336). The response rate was 77% in London and 71% in Birmingham. In the London sample, 73% reported current smoking and 23% reported drinking alcohol every day in the past week. The corresponding values in the housed group were 27% and 9%. Table 1 summarises the characteristics of the study population.

<< Table 1 about here (see end of manuscript) >>

Long-term conditions

The prevalence of any long-term condition was 34.2% (95% CI 31.6%, 36.8%) in the homeless group and 12.1% (95% CI 11.5%, 12.6%) in the housed group. Asthma was the most commonly reported condition in both groups. All conditions except for diabetes were more common in the homeless group than in the housed group. COPD, heart problems, stroke and diabetes were strongly associated with older age for both homeless and housed groups, while epilepsy and asthma were not (figure 1 and Supplementary Information).

<< figure 1 about here: Figure 1: Percentage reporting long-term conditions, by age group >>

When we compared long-term conditions across deprivation quintiles in the housed population, we observed social gradients for all diseases except asthma ($p<0.05$), with participants living in deprived areas having higher risk (figure 2). For all conditions except diabetes, there were larger relative and absolute differences between the homeless group and all housed deprivation groups than there were between the housed deprivation groups. For example, the prevalence ratio comparing the risk of COPD in the most deprived quintile of housed participants versus the least deprived was 1.8 (95% CI 1.2, 2.9), compared to 12.9 (95% CI 8.8, 19.0) comparing the homeless group to the least deprived quintile.

<< figure 2 about here: Figure 2: Prevalence of long-term conditions (top panel) and prevalence ratios (bottom panel), with 95% confidence intervals >>

Health-related quality of life (EQ-5D-3L)

The homeless group reported worse overall health, with a median of 65 (IQR 50-80) on the EQ-5D-3L VAS, compared to median of 80 (IQR 70-90) for the housed group (figure 3).

<< figure 3 about here: Figure 3: Histograms of EQ5D-Visual Analogue Scale score, by homeless/housed status. Horizontal lines show median and 25% and 75% quantiles. >>

For peer review only - http://bmjopen.bmj.com/site/about/guidelines.xhtml

802/1194 (67%) of the homeless group reported problems in at least one of the EQ-5D-3L domains (mobility, self-care, usual activities, pain and anxiety), compared to 4292/13360 (35.7%) of the housed group. The homeless group reported more problems than the housed group in all five domains.

When we compared EQ-5D-3L problems across deprivation quintiles in the housed population, we observed social gradients for all domains ($p<0.0001$), with participants living in deprived areas more likely to report problems (figure 4). In contrast to the results for disease prevalence, the homeless group and the most deprived housed quintile had similar likelihood of reporting problems related to mobility, self-care, usual activities and pain. For example, the prevalence of problems with usual activities in the most deprived quintile of housed participants was 2.5 times the least deprived group (95% CI 2.1, 2.9), compared to 2.2 (95% CI 1.8, 2.7) for the homeless group. However, the homeless group had substantially greater likelihood of reporting problems with anxiety than all housed groups. The most deprived quintile of housed participants was 1.8 times more likely to report problems with anxiety than the least deprived group (95% CI 1.6, 2.0), compared to 3.8 (95% CI 3.4, 4.2) for the homeless group.

The joint effects analysis (Supplementary Information) of morbidity and deprivation showed that greater deprivation was associated with higher likelihood of reporting problems in all EQ-5D-3L domains, for participants both with and without long-term conditions. After accounting for morbidity, homeless participants had similar or lower likelihood of reporting problems in all domains except anxiety, when compared to than the most deprived housed participants.

<< figure 4 about here: Figure 4: Health-related quality of life: prevalence of problems by deprivation group (top panel) and prevalence ratios (bottom panel), with 95% confidence intervals >>

For peer review only - http://bmjopen.bmj.com/site/about/guidelines.xhtml

DISCUSSION

Principle findings

Homeless people report substantially worse health than those in stable housing and are three times more likely to report a chronic disease. In particular, homeless people report higher prevalence of asthma, COPD, epilepsy and heart problems. Homeless people also report worse quality of life, and are more than twice as likely to report problems with anxiety. There are large differences between the homeless group and the most deprived housed group. When compared to the 'slopes' in health outcomes across deprivation quintiles, the inequalities in outcomes for homeless people appear more like a 'cliff'.

Limitations

This is a cross-sectional study and does not provide insight into why homeless people have worse health than housed people. We aimed to describe the prevalence of health problems rather than understand these causal pathways, and therefore did not attempt to control for factors such as health behaviours.

EQ-5D-3L has not been validated in homeless people. However, it has been used in previous studies of the health of homeless people [22] and validity has been demonstrated in overlapping groups such as people who use illicit drugs [23]. There may be value in future research into the interpretation of quality of life instruments by homeless people.

Comparisons with existing studies

The health of homeless people differs between countries and regions. However, the large inequalities between homeless and housed people are likely to persist. Studies that applied EQ-5D-3L to 73 homeless people in Italy [24] and 155 in Sweden [22] both found substantially more problems related to anxiety than other domains, reflecting our results. While there are few studies of long-term conditions in homeless people, existing studies have also found high frequency of non-communicable diseases [11–15].

Implications

There are many possible causes of the 'cliff' in long-term conditions and anxiety observed for homeless people, and the association between homelessness and poor health is most likely bidirectional. Poor health may precipitate homelessness if it leads to loss of income or breakdown of relationships. This is supported by data from Scotland showing high rates of health service use before first episodes of homelessness [25]. Homelessness is also likely to worsen physical health due to poor living conditions; use of tobacco, alcohol and drugs [11]; and poor access to health services [26].

Despite the higher prevalence of long-term conditions, homeless people did not report substantially more quality-of-life problems (apart from problems related to anxiety).

For peer review only - http://bmjopen.bmj.com/site/about/guidelines.xhtml

This may reflect a higher threshold for reporting problems, due to a low expectations of health or normalisation of pain and illness.

The age of onset of long-term conditions is related to the patterns observed across age groups (figure 1). The prevalence of asthma and epilepsy is flat across age groups in both the housed and homeless populations. These diseases typically onset in childhood and the higher prevalence in the homeless group may relate to circumstances in childhood or before homelessness. For example, heavy alcohol use and brain injury [27] are common precursors of homelessness and may contribute to higher prevalence of epilepsy. COPD and heart problems are strongly associated with adult risk factors, particularly smoking, which reflects the higher prevalence observed in older age groups, and the higher prevalence in the homeless group. The average age of first stroke is 71 (sd. 13) in men in the UK [28], which is older than the study population, reflecting the small number of cases and limited power to observe inequalities, though higher prevalence was still observed in the homeless group (table 1).

Research into the health of homeless people has focused on infections, mental health and ‘external’ causes such as drug overdose. Evaluations of interventions have also focused on these areas. For example, there is evidence that case management can improve the effectiveness of treatments for mental health problems, drug dependence and tuberculosis in homeless people [29]. Research into 'housing first', an approach that provides housing before engaging individuals in treatment for substance use or mental health problems, also looks at how it can improve outcomes in these areas [30]. The focus on these areas may have resulted from the high relative risks of disease [7], in part due to their rarity in the general population, and because in most studies of mortality they account for the majority of deaths.

Our results show that respiratory diseases, epilepsy and heart problems are also common among homeless people in England. Homeless people encounter barriers to management of chronic diseases [31] and there is a need for research into effective healthcare. Case management should be further explored for management of chronic diseases. Existing evidence-based preventative interventions should also be adapted and evaluated for excluded groups. For example, smoking cessation interventions can reduce the risk of COPD and improve asthma symptoms, and should be tailored to homeless people and evaluated. There is also a need for routine surveillance data on the health of homeless people, to improve transparency and accountability for poor health in this population.

Conclusion

There is a ‘slope’ in health outcomes across socioeconomic groups in the general population and a ‘cliff’ when we consider homeless people. These extreme differences extend to long-term physical health conditions as well as infections and mental health.

For peer review only - http://bmjopen.bmj.com/site/about/guidelines.xhtml

TABLE

Table 1: characteristics of study population

		Find & Treat (homeless)	Health Survey for England (housed)	p-value
Sample size		1336	13360	
Age group	16-24	140 (10.5)	1400 (10.5)	*Matched*
	25-34	364 (27.2)	3640 (27.2)	
	35-44	374 (28.0)	3740 (28.0)	
	45-54	315 (23.6)	3150 (23.6)	
	55-64	143 (10.7)	1430 (10.7)	
Sex	Male	249 (18.6)	2490 (18.6)	*Matched*
	Female	1087 (81.4)	10870 (81.4)	
Current smoker	Yes	317 (23.7)	3463 (25.9)	*
	No	115 (8.6)	9839 (73.6)	
	Missing	904 (67.7)	58 (0.4)	
Drank alcohol every day last week	Yes	101 (7.6)	1087 (8.1)	*
	No	331 (24.8)	12271 (91.8)	
	Missing	904 (67.7)	2 (0.01)	
Injects drugs	Current	82 (6.1)	-	*
	Past	191 (14.3)	-	
	Never	1034 (77.4)	-	
	Missing	29 (2.2)	13360 (100.0)	
Prevalence of long-term conditions	Asthma	244 (18.3)	756 (5.7)	p<0.001
	COPD	187 (14.0)	180 (1.3)	p<0.001
	Epilepsy	80 (6.0)	108 (0.8)	p<0.001
	Heart problems	103 (7.7)	266 (2.0)	p<0.001
	Stroke	24 (1.8)	53 (0.4)	p<0.001
	Diabetes	55 (4.1)	431 (3.2)	p=0.098
**EQ-5D-3L (health-related quality of life)**				
Reporting problems**	Mobility	251 (21.0)	1192 (9.9)	p<0.001
	Self-care	92 (7.7)	377 (3.1)	p<0.001
	Usual activities	178 (14.9)	1261 (10.5)	p<0.001
	Pain	432 (36.2)	3034 (25.3)	p<0.001
	Anxiety	646 (54.1)	2229 (18.6)	p<0.001
Visual analogue scale	Median (IQR)	65 (50-80)	80 (70-90)	p<0.001
	Score < 75	676 (63.8)	1721 (29.3)	p<0.001

* Differences in health behaviours are not tested due to missing data.
**The proportions exclude 142 cases in the Find & Treat group and 1345 cases in the Health Survey for England group who did not answer all EQ-5D-3L questions

For peer review only - http://bmjopen.bmj.com/site/about/guidelines.xhtml

ADDITIONAL INFORMATION

Funding

Dan Lewer is funded by an NIHR Doctoral Research Fellowship (DRF-2018-11-ST2-016). Robert W Aldridge is funded by a Wellcome Trust Clinical Research Career Development Fellowship (206602/Z/17/Z). Imtiaz Ahmed is funded by Sandwell Hospital and Public Health England. Serena A Luchenski is funded by an NIHR Clinical Doctoral Research Fellowship (ICA-CDRF-2016-02-042).

Contributions

AS and AH conceived of the initial idea. DL, RWA, DM, CS, PZ, MJD, IA, SL, AH and AS contributed to the design of the study. DL conducted data analysis and wrote the first draft of the paper. DL, RWA, DM, CS, PZ, MJD, IA, SL, AH and AS contributed to the main content of the manuscript, provided comments on the final draft, and approved the manuscript before submission.

Competing interests

AH is Trustee of the UK-based charity 'Pathway (healthcare for homeless people)'. AS is Clinical Lead for the Find & Treat Service; data was collected from homeless clients of this service.

Data availability

Researchers can obtain anonymised survey data from the Find and Treat service at University College London Hospitals NHS Foundation Trust. Please contact al.story@nhs.net for further information.

For peer review only

For peer review only - http://bmjopen.bmj.com/site/about/guidelines.xhtml

REFERENCES

1 Ministry of Housing, Communities & Local Government. Rough Sleeping Statistics Autumn 2017, England (Revised). 2017. https://assets.publishing.service.gov.uk/government/uploads/system/uploads/attachment_data/file/682001/Rough_Sleeping_Autumn_2017_Statistical_Release_-_revised.pdf (accessed 18 Dec 2018).

2 Tsai J. Lifetime and 1-year prevalence of homelessness in the US population: results from the National Epidemiologic Survey on Alcohol and Related Conditions-III. *Journal of Public Health* 2018;**40**:65–74. doi:10.1093/pubmed/fdx034

3 Morrison DS. Homelessness as an independent risk factor for mortality: results from a retrospective cohort study. *International Journal of Epidemiology* 2009;**38**:877–83. doi:10.1093/ije/dyp160

4 Stenius-Ayoade A, Haaramo P, Kautiainen H, *et al.* Mortality and causes of death among homeless in Finland: a 10-year follow-up study. *Journal of Epidemiology and Community Health* 2017;**71**:841–8. doi:10.1136/jech-2017-209166

5 Nielsen SF, Hjorthøj CR, Erlangsen A, *et al.* Psychiatric disorders and mortality among people in homeless shelters in Denmark: a nationwide register-based cohort study. *The Lancet* 2011;**377**:2205–14. doi:10.1016/S0140-6736(11)60747-2

6 Nordentoft M. 10 year follow up study of mortality among users of hostels for homeless people in Copenhagen. *BMJ* 2003;**327**:81–0. doi:10.1136/bmj.327.7406.81

7 Fugelstad A, Beijer U, Andreasson S, *et al.* Mortality and causes of death among homeless women and men in Stockholm. *Scandinavian Journal of Public Health* 2011;**39**:121–7. doi:10.1177/1403494810393554

8 Office for National Statistics. Deaths of homeless people in England and Wales. 2018.https://www.ons.gov.uk/peoplepopulationandcommunity/birthsdeathsandmarriages/deaths/bulletins/deathsofhomelesspeopleinenglandandwales/2013to2017 (accessed 18 Dec 2018).

9 Fazel S, Geddes JR, Kushel M. The health of homeless people in high-income countries: descriptive epidemiology, health consequences, and clinical and policy recommendations. *Lancet* 2014;**384**:1529–40. doi:10.1016/S0140-6736(14)61132-6

10 Aldridge RW, Story A, Hwang SW, *et al.* Morbidity and mortality in homeless individuals, prisoners, sex workers, and individuals with substance use disorders in high-income countries: a systematic review and meta-analysis. *The Lancet* 2018;**391**:241–50. doi:10.1016/S0140-6736(17)31869-X

For peer review only - http://bmjopen.bmj.com/site/about/guidelines.xhtml

11 Homeless Link. The unhealthy state of homelessness: Health audit results. 2014.http://www.homeless.org.uk/sites/default/files/site-attachments/The unhealthy state of homelessness FINAL.pdf (accessed 27 Jun 2018).

12 Keogh C, O'Brien KK, Hoban A, *et al.* Health and use of health services of people who are homeless and at risk of homelessness who receive free primary health care in Dublin. *BMC health services research* 2015;**15**:58. doi:https://dx.doi.org/10.1186/s12913-015-0716-4

13 Brett T, Arnold-Reed DE, Troeung L, *et al.* Multimorbidity in a marginalised, street-health Australian population: a retrospective cohort study. *BMJ Open* 2014;**4**:e005461–e005461. doi:10.1136/bmjopen-2014-005461

14 Bines W. *The health of single homeless people*. York: : Centre for Housing Policy, University of York 1994.

15 Beijer U, Bruce D, Burström B. Changes over time in the risk of hospitalization for physical diseases among homeless men and women in Stockholm: A comparison of two cohorts. *Scandinavian Journal of Public Health* 2016;**44**:784–90. doi:10.1177/1403494816671601

16 Mackenbach J, Kunst A, Cavelaars A, *et al.* Socioeconomic inequalities in morbidity and mortality in western Europe. *Lancet* 1997;**349**:1655–9. doi:10.1016/S0140-6736(96)07226-1

17 Marmot MG, Stansfeld S, Patel C, *et al.* Health inequalities among British civil servants: the Whitehall II study. *The Lancet* 1991;**337**:1387–93. doi:10.1016/0140-6736(91)93068-K

18 Craig R, Fuller E, Mindell J. Health Survey for England 2014, Volume 2: Methods and Documentation. 2014.https://files.digital.nhs.uk/publicationimport/pub19xxx/pub19295/hse2014-methods-and-docs.pdf (accessed 27 Jun 2018).

19 The EuroQol Group. EuroQol - a new facility for the measurement of health-related quality of life. *Health policy* 1990;**16**:199–208. doi:10.1016/0168-8510(90)90421-9

20 DCLG. The English Indices of Deprivation 2010. Neighbourhoods Statistical Release. 2011.https://assets.publishing.service.gov.uk/government/uploads/system/uploads/attachment_data/file/6320/1870718.pdf (accessed 27 Jun 2018).

21 NHS Health Research Authority, Medical Research Council. Do I need NHS REC approval? http://www.hra-decisiontools.org.uk/ethics/ (accessed 1 Dec 2017).

22 Sun S, Irestig R, Burström B, *et al.* Health-related quality of life (EQ-5D) among homeless persons compared to a general population sample in Stockholm County, 2006. *Scandinavian Journal of Social Medicine* 2012;**40**:115–25. doi:10.1177/1403494811435493

For peer review only - http://bmjopen.bmj.com/site/about/guidelines.xhtml

23 Goranitis I, Coast J, Day E, *et al.* Measuring Health and Broader Well-Being Benefits in the Context of Opiate Dependence: The Psychometric Performance of the ICECAP-A and the EQ-5D-5L. *Value in Health* 2016;**19**:820–8. doi:10.1016/j.jval.2016.04.010

24 Levorato S, Bocci G, Troiano G, *et al.* Health status of homeless persons: a pilot study in the Padua municipal dorm. *Annali di igiene : medicina preventiva e di comunita* 2017;**29**:54–62. doi:https://dx.doi.org/10.7416/ai.2017.2132

25 Waugh A, Clarke A, Knowles J, *et al.* Health and Homelessness in Scotland. 2018.https://www.gov.scot/publications/health-homelessness-scotland/ (accessed 18 Dec 2018).

26 Palepu A, Gadermann A, Hubley AM, *et al.* Substance Use and Access to Health Care and Addiction Treatment among Homeless and Vulnerably Housed Persons in Three Canadian Cities. *PLoS ONE* 2013;**8**. doi:10.1371/journal.pone.0075133

27 Topolovec-Vranic J, Ennis N, Colantonio A, *et al.* Traumatic brain injury among people who are homeless: A systematic review. *BMC Public Health* 2012;**12**. doi:10.1186/1471-2458-12-1059

28 Lee S, Shafe ACE, Cowie MR. UK stroke incidence, mortality and cardiovascular risk management 1999-2008: time-trend analysis from the General Practice Research Database. *BMJ Open* 2011;**1**:e000269–e000269. doi:10.1136/bmjopen-2011-000269

29 Hwang SW, Tolomiczenko G, Kouyoumdjian FG, *et al.* Interventions to Improve the Health of the Homeless: A Systmatic Review. *American Journal of Preventive Medicine* 2005;**29**:311.e1-311.e9. doi:10.1016/j.amepre.2005.06.017

30 Woodhall-Melnik JR, Dunn JR. A systematic review of outcomes associated with participation in Housing First programs. *Housing Studies* 2016;**31**:287–304. doi:10.1080/02673037.2015.1080816

31 Hwang SW, Bugeja AL. Barriers to appropriate diabetes management among homeless people in Toronto. *CMAJ : Canadian Medical Association journal = journal de l'Association medicale canadienne* 2000;**163**:161–5.

For peer review only - http://bmjopen.bmj.com/site/about/guidelines.xhtml

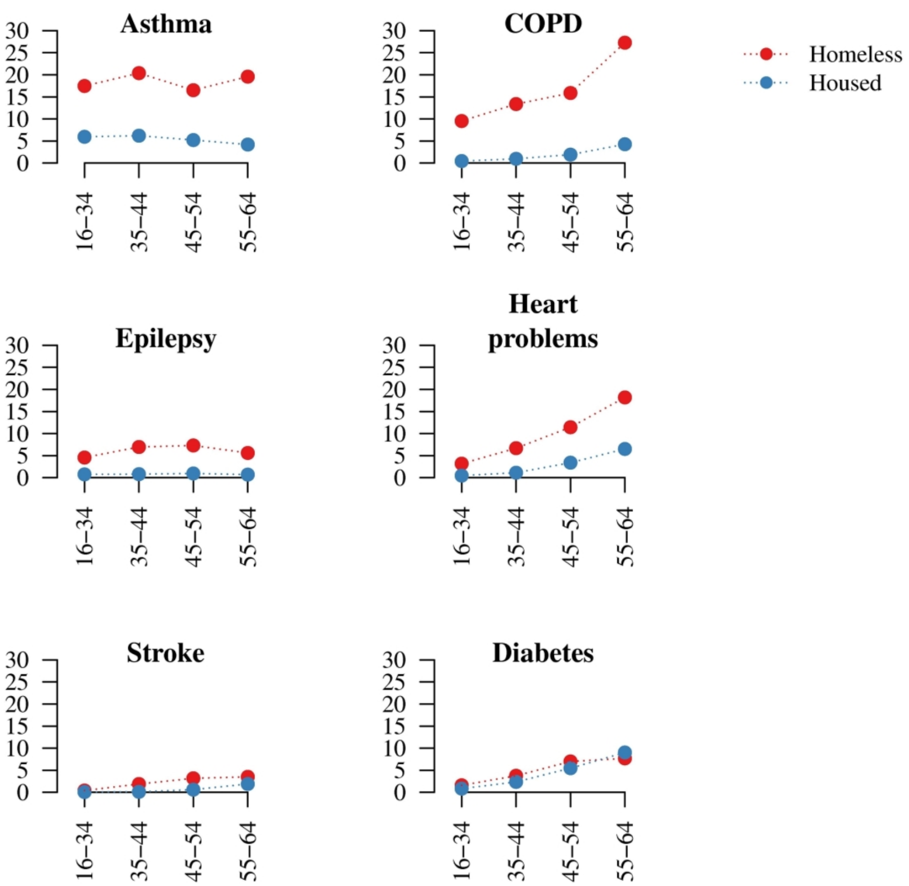

Figure 1: Percentage reporting long-term conditions, by age group

177x177mm (300 x 300 DPI)

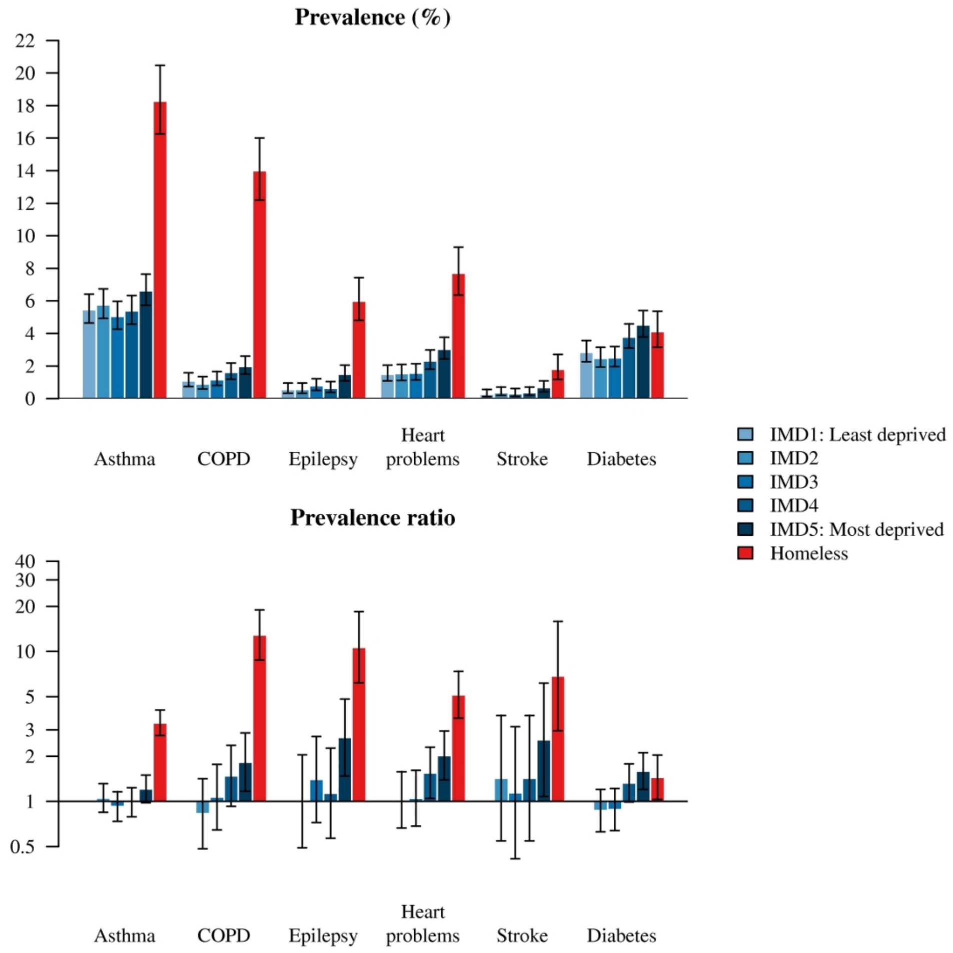

Figure 2: Prevalence of long-term conditions (top panel) and prevalence ratios (bottom panel), with 95% confidence intervals

177x177mm (300 x 300 DPI)

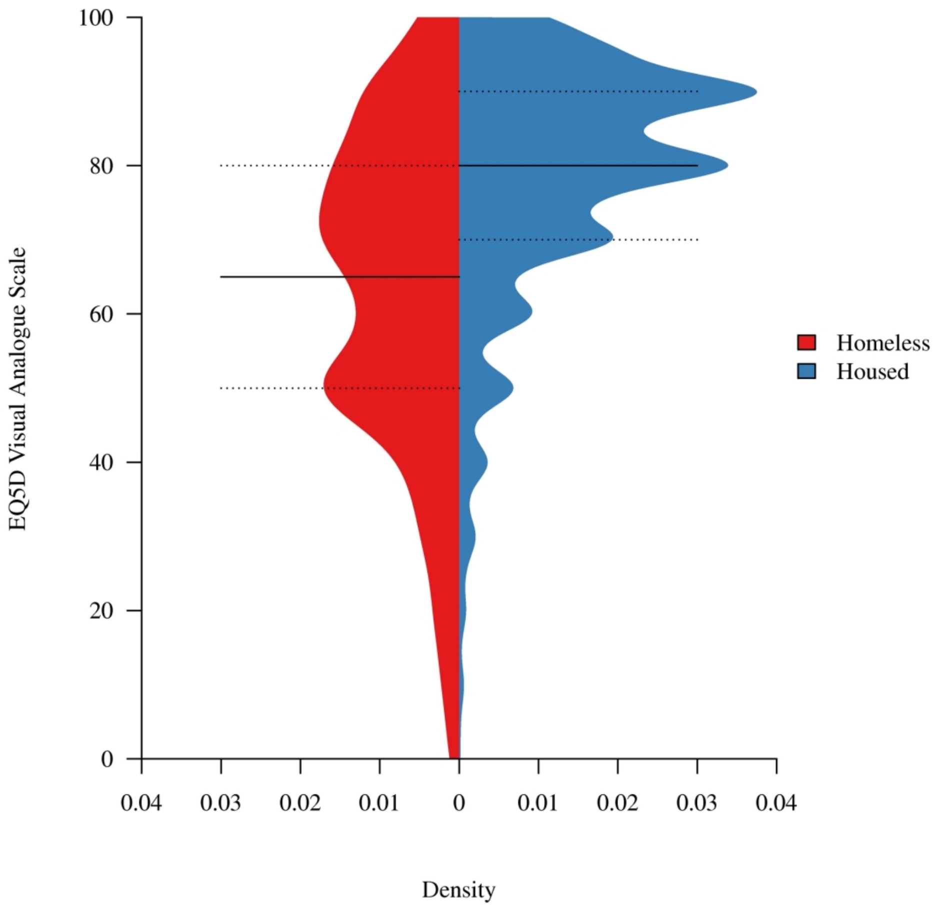

Figure 3: Histograms of EQ5D-Visual Analogue Scale score, by homeless/housed status. Horizontal lines show median and 25% and 75% quantiles

177x177mm (300 x 300 DPI)

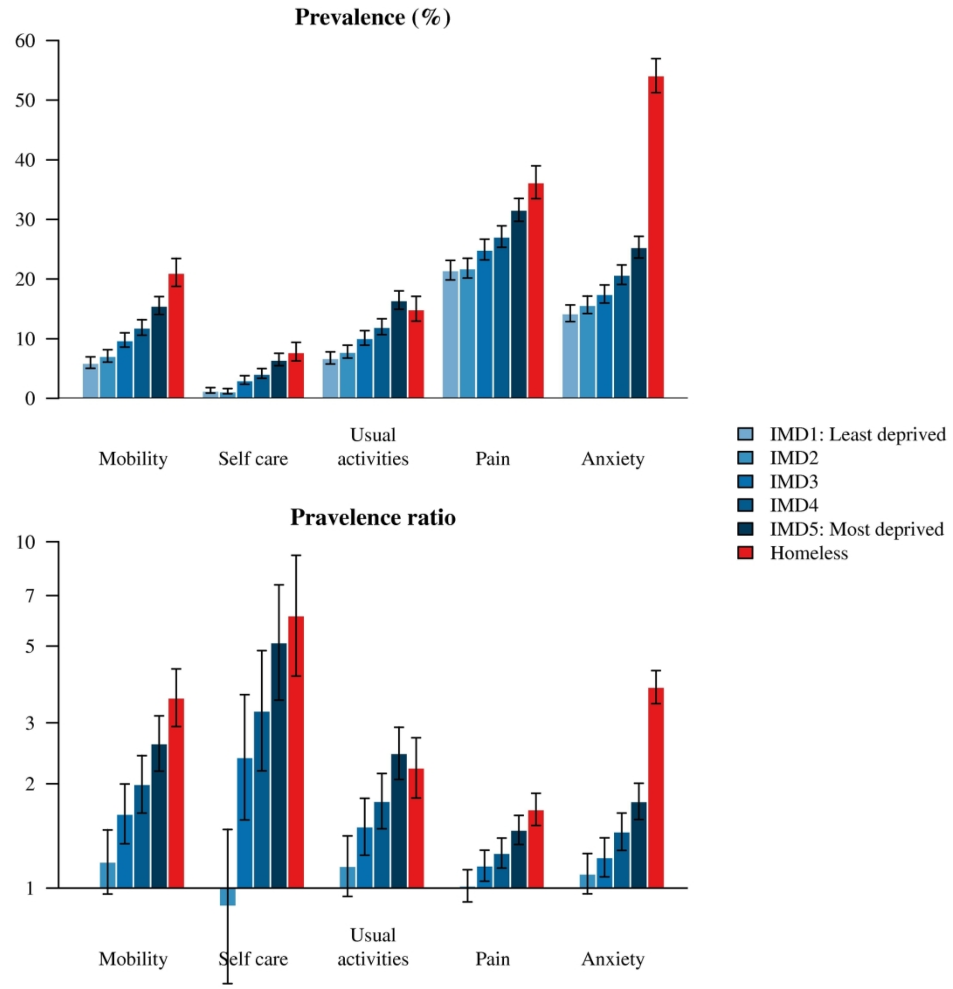

Figure 4: Health-related quality of life: prevalence of problems by deprivation group (top panel) and prevalence ratios (bottom panel), with 95% confidence intervals

177x177mm (300 x 300 DPI)

Supplementary information

1. Table S1: prevalence of long term conditions by age group
2. Figure. Health-related quality of life in participants with one or more long-term condition
3. Table S2: Sensitivity analysis excluding Health Survey for England data 2006 and 2008

For peer review only

For peer review only - http://bmjopen.bmj.com/site/about/guidelines.xhtml

1. Table S1. prevalence of long-term condition by age group: cases/sample size (prevalence; 95% confidence intervals). Stroke is excluded to avoid publication of small numbers

Condition	Age group	Find & Treat (homeless)	Health Survey for England (housed)
Asthma	16-34	88/504 (17.5; 14.3-21.1)	300/5040 (6.0; 5.3-6.7)
	35-44	76/373 (20.4; 16.5-24.9)	232/3740 (6.2; 5.5-7.0)
	45-54	52/315 (16.5; 12.7-21.2)	164/3150 (5.2; 4.5-6.1)
	55-64	28/143 (19.6; 13.6-27.2)	60/1430 (4.2; 3.2-5.4)
		p=0.542	p=0.020
COPD	16-34	48/504 (9.5; 7.2-12.5)	23/5040 (0.5; 0.3-0.7)
	35-44	50/374 (13.4; 10.2-17.3)	36/3740 (1.0; 0.7-1.3)
	45-54	50/315 (15.9; 12.1-20.5)	60/3150 (1.9; 1.5-2.5)
	55-64	39/143 (27.3; 20.3-35.5)	61/1430 (4.3; 3.3-5.5)
		p<0.001	p<0.001
Epilepsy	16-34	23/504 (4.6; 3.0-6.9)	38/5040 (0.8; 0.5-1.0)
	35-44	26/374 (7.0; 4.7-10.1)	30/3740 (0.8; 0.6-1.2)
	45-54	23/315 (7.3; 4.8-10.9)	30/3150 (1.0; 0.7-1.4)
	55-64	8/143 (5.6; 2.6-11.1)	10/1430 (0.7; 0.4-1.3)
		p=0.329	p=0.749
Heart problems	16-34	16/504 (3.2; 1.9-5.2)	24/5040 (0.5; 0.3-0.7)
	35-44	25/374 (6.7; 4.5-9.8)	42/3740 (1.1; 0.8-1.5)
	45-54	36/315 (11.4; 8.2-15.6)	107/3150 (3.4; 2.8-4.1)
	55-64	26/143 (18.2; 12.4-25.7)	93/1430 (6.5; 5.3-7.9)
		p<0.001	p<0.001
Diabetes	16-34	8/504 (1.6; 0.7-3.2)	42/5040 (0.8; 0.6-1.1)
	35-44	14/374 (3.7; 2.1-6.3)	88/3740 (2.4; 1.9-2.9)
	45-54	22/315 (7.0; 4.5-10.5)	172/3150 (5.5; 4.7-6.3)
	55-64	11/143 (7.7; 4.1-13.7)	129/1430 (9.0; 7.6-10.7)
		p<0.001	p<0.001

Age groups 16-24 and 25-34 have been merged to avoid publication of small numbers. p-values are based on chi-square tests.

For peer review only - http://bmjopen.bmj.com/site/about/guidelines.xhtml

For peer review only

2. Figure S1. Odds ratios of reported problems in EQ-5D-3L domains (some and severe problems grouped), by deprivation and presence of any long-term conditions, with 95% confidence intervals

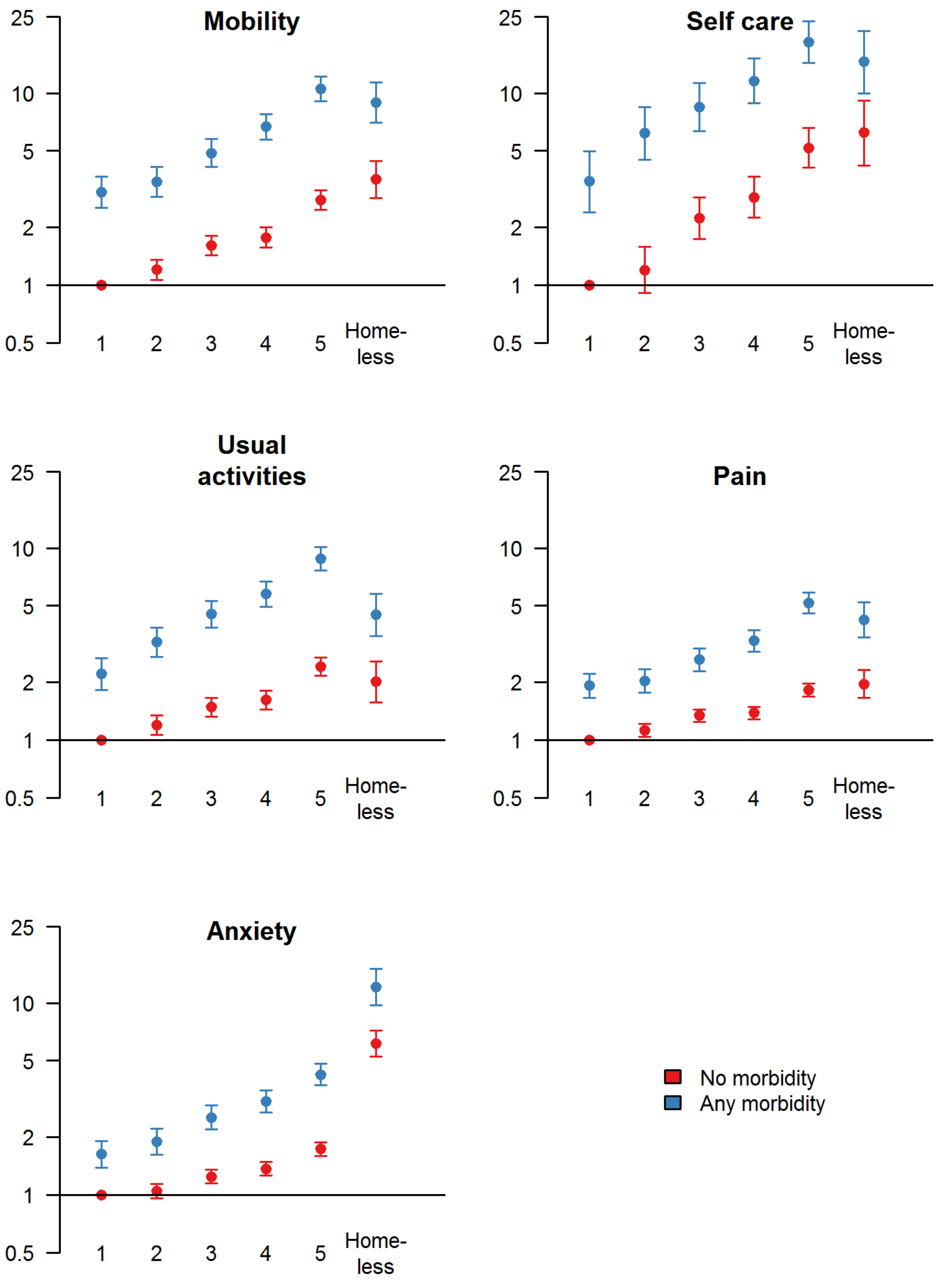

x-axis show deprivation groups (1 = least deprived quintile of Index of Multiple Deprivation; 5 = most deprived quintile of Index of Multiple deprivation). Y-axis shows odds ratios, with least deprived group with no morbidity as the reference group.

3. Table S2: Sensitivity analysis excluding Health Survey for England data 2006 and 2008: prevalence ratio comparing homeless participants to the least deprived housed quintile

		Original analysis	Excluding HSE data from 2006 and 2008
**Long-term conditions**	Asthma	3.3 (2.8-4.1)	3.7 (2.8-4.7)
	Breathing	12.9 (8.8-19.0)	16.6 (9.3-29.5)
	Epilepsy	10.7 (6.2-18.4)	12.1 (5.6-26.2)
	Heart problems	5.1 (3.6-7.4)	6.8 (4.1-11.5)
	Stroke	6.9 (3.0-15.9)	8.5 (2.6-28.2)
	Diabetes	1.4 (1.0-2.0)	1.6 (1.1-2.5)
**EQ-5D problems**	Mobility	3.5 (2.9-4.3)	3.3 (2.6-4.2)
	Self-care	6.1 (4.1-9.1)	7.6 (4.3-13.5)
	Usual activities	2.2 (1.8-2.7)	2.0 (1.6-2.5)
	Pain	1.7 (1.5-1.9)	1.6 (1.4-1.8)
	Anxiety	3.8 (3.4-4.2)	3.2 (2.8-3.7)

For peer review only - http://bmjopen.bmj.com/site/about/guidelines.xhtml

STROBE Statement—Checklist of items that should be included in reports of ***cross-sectional studies***

	Item No	Recommendation	Page
**Title and abstract**	1	(*a*) Indicate the study's design with a commonly used term in the title or the abstract	1
		(*b*) Provide in the abstract an informative and balanced summary of what was done and what was found	2
**Introduction**			
Background/rationale	2	Explain the scientific background and rationale for the investigation being reported	4
Objectives	3	State specific objectives, including any prespecified hypotheses	4
**Methods**			
Study design	4	Present key elements of study design early in the paper	5-6
Setting	5	Describe the setting, locations, and relevant dates, including periods of recruitment, exposure, follow-up, and data collection	4
Participants	6	(*a*) Give the eligibility criteria, and the sources and methods of selection of participants	4
Variables	7	Clearly define all outcomes, exposures, predictors, potential confounders, and effect modifiers. Give diagnostic criteria, if applicable	5-6
Data sources/ measurement	8*	For each variable of interest, give sources of data and details of methods of assessment (measurement). Describe comparability of assessment methods if there is more than one group	5-6
Bias	9	Describe any efforts to address potential sources of bias	5-6
Study size	10	Explain how the study size was arrived at	N/A
Quantitative variables	11	Explain how quantitative variables were handled in the analyses. If applicable, describe which groupings were chosen and why	5-6
Statistical methods	12	(*a*) Describe all statistical methods, including those used to control for confounding	6
		(*b*) Describe any methods used to examine subgroups and interactions	6
		(*c*) Explain how missing data were addressed	6
		(*d*) If applicable, describe analytical methods taking account of sampling strategy	N/A
		(*e*) Describe any sensitivity analyses	6
**Results**			
Participants	13*	(a) Report numbers of individuals at each stage of study—eg numbers potentially eligible, examined for eligibility, confirmed eligible, included in the study, completing follow-up, and analysed	N/A
		(b) Give reasons for non-participation at each stage	N/A
		(c) Consider use of a flow diagram	N/A
Descriptive data	14*	(a) Give characteristics of study participants (eg demographic, clinical, social) and information on exposures and potential confounders	12
		(b) Indicate number of participants with missing data for each variable of interest	6,12
Outcome data	15*	Report numbers of outcome events or summary measures	8,9,12
Main results	16	(*a*) Give unadjusted estimates and, if applicable, confounder-adjusted estimates and their precision (eg, 95% confidence interval). Make clear which confounders were adjusted for and why they were included	8,9,12

For peer review only

		(*b*) Report category boundaries when continuous variables were categorized	5-6
		(*c*) If relevant, consider translating estimates of relative risk into absolute risk for a meaningful time period	N/A
Other analyses	17	Report other analyses done—eg analyses of subgroups and interactions, and sensitivity analyses	Supp.
**Discussion**			
Key results	18	Summarise key results with reference to study objectives	10
Limitations	19	Discuss limitations of the study, taking into account sources of potential bias or imprecision. Discuss both direction and magnitude of any potential bias	10
Interpretation	20	Give a cautious overall interpretation of results considering objectives, limitations, multiplicity of analyses, results from similar studies, and other relevant evidence	10-11
Generalisability	21	Discuss the generalisability (external validity) of the study results	11
**Other information**			
Funding	22	Give the source of funding and the role of the funders for the present study and, if applicable, for the original study on which the present article is based	13

*Give information separately for exposed and unexposed groups.

Note: An Explanation and Elaboration article discusses each checklist item and gives methodological background and published examples of transparent reporting. The STROBE checklist is best used in conjunction with this article (freely available on the Web sites of PLoS Medicine at http://www.plosmedicine.org/, Annals of Internal Medicine at http://www.annals.org/, and Epidemiology at http://www.epidem.com/). Information on the STROBE Initiative is available at www.strobe-statement.org.

For peer review only
